# Perfluoro(2-ethoxy-2-fluoroethoxy)-acetic Acid and Other Target and Suspect PFAS in the Vicinity of a Fluoropolymer Production Plant

**DOI:** 10.1021/acs.est.5c07856

**Published:** 2025-07-18

**Authors:** Joost Dalmijn, Jonathan P. Benskin, Matthew E. Salter, Andrew J. Sweetman, Crispin J. Halsall, Jack Garnett, Ian T. Cousins

**Affiliations:** † Department of Environmental Science, 7675 Stockholm University, SE-10691 Stockholm, Sweden; ‡ Lancaster Environment Centre, 4396 Lancaster University, LA1 4YQ Lancaster, U.K.

**Keywords:** suspect screening, EEA, PTFE, air sampling, surface water sampling, byproducts, high-resolution mass spectrometry

## Abstract

Multiple target and suspect per- and polyfluoroalkyl substances (PFAS), including the replacement fluorinated processing aid perfluoro­(2-ethoxy-2-fluoroethoxy)-acetic acid (“EEA”), were measured in both air and surface water in the vicinity of a fluoropolymer production plant (FPP) in Thornton-Cleveleys (United Kingdom) during sampling campaigns in 2021 and 2023, respectively. Targeted and suspect screening methods were conducted using ultrahigh-performance liquid chromatography (UHPLC) coupled with Q-Exactive HF Orbitrap high-resolution mass spectrometry (HRMS). Summed PFAS levels in water nearby the plant ranged from 30 to 22,542 ng/L and were dominated by perfluoroalkyl carboxylic acids (PFCAs) and perfluoroalkyl ether carboxylic acids (PFECAs), most notably perfluorooctanoic acid (PFOA; up to 20,624 ng/L), EEA (up to 1744 ng/L), H-PFOA (up to 1027 ng/L), and perfluorohexanoic acid (PFHxA; up to 650 ng/L). Additionally, various homologous series of PFAS suspects, such as hydrogen-substituted PFCAs (H-PFCAs), chlorine-substituted PFCAs (Cl-PFCAs), and monoether perfluoroether alkyl carboxylic acids (ME-PFECAs) were identified, some for the first time in Europe. In air, PFOA was detected in all but one sample collected 20 km from the plant at concentrations ranging from 0.51 to 2.50 pg/m^3^. The three air samples that showed detectable EEA concentrations also displayed evidence of long-chain targets and suspects and were associated with high wind speeds from a southwesterly direction. Overall, this study shows that this site continues to be a source of a complex mixture of legacy and scarcely monitored PFAS that occur in multiple environmental media. This highlights the importance of further research that assesses the toxicity of these substances and the resulting impacts on humans and wildlife.

## Graphical abstract

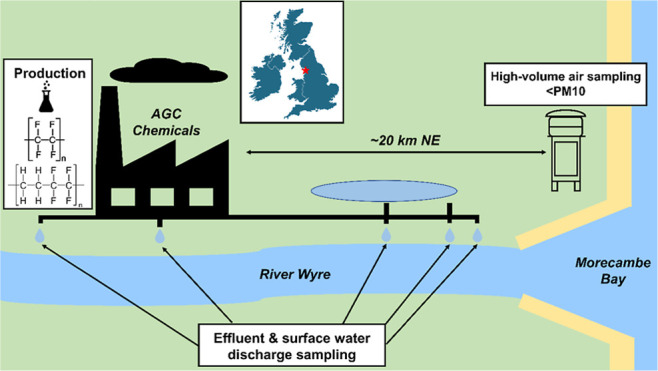

## 1 Introduction

Fluoropolymers are a subset of per- and polyfluoroalkyl substances (PFAS) consisting of a carbon–fluorine backbone that are produced through the polymerization of low-molecular weight fluorinated organic monomers. Well-known fluoropolymers include polytetrafluoroethylene (PTFE), polyvinylidene fluoride (PVDF), and fluoroelastomers (FKMs).^1,2^ Owing to the presence of multiple carbon–fluorine bonds, these specialty materials have various useful and unique properties, such as high thermal and chemical resistance and low surface energy.^3^ As a result, fluoropolymers are widely used in many different industrial and consumer applications where these properties are desired.^4^ Fluoropolymers are the second-largest subgroup of PFAS globally with several hundred thousand tons produced annually and second only to fluorinated gases.^5^

A wide variety of PFAS and other fluorinated organic substances are used and/or formed during the production of fluoropolymers.^6^ Fluorosurfactants, which consist of a hydrophobic per- or polyfluoroalkyl chain and a hydrophilic headgroup (e.g., carboxylate or sulfonate), are among the most well-known substances associated with fluoropolymer production.^7^ Fluorosurfactant salts (e.g., with ammonium, sodium, or potassium counterions) are used as processing aids that serve to stabilize and disperse aqueous emulsions of monomers in the emulsion polymerization process. Additionally, fluorosurfactants can be formed as byproducts during fluoropolymer production.^8,9^ Because these substances are not part of the polymer end-product, producers attempt to remove them via various processing steps. Liquid and gaseous waste streams of fluoropolymer production plants are known to contain residual fluorosurfactants.^9,54^ If left untreated, such streams can cause these sites to act as point sources of these PFAS to the environment.

While PFAS fluorosurfactants play an important role in the emulsion polymerization process, other processes that are employed in fluoropolymer production such as suspension polymerization, the synthesis of fluoromonomers through pyrolysis, and further processing steps such as polymer cross-linking and molecular weight adjustment also involve the use or formation of PFAS or fluorinated organic substances. Examples of these substances include fluorinated monomers, pyrolysis byproducts, fluorinated solvents, oligomers, chain transfer agents, and cross-linking agents.^6^

When considering PFAS emissions, unchecked releases of the long-chain perfluoroalkyl carboxylic acid (PFCA) fluorosurfactant processing aids perfluorooctanoic acid (PFOA) and perfluorononanoic acid (PFNA) by the fluoropolymer production industry from the 1950s until the early 2010s have contributed significantly to global levels of these substances.^7^ Following concerns about their persistence, bioaccumulation potential, and toxicity, these long-chain fluorosurfactants were voluntarily phased out as processing aids in Europe, Japan, and the USA between 2002 and 2016. Various replacement substances have since emerged. Many of these replacement fluorosurfactants are proprietary ammonium salts of perfluoroalkyl ether carboxylic acids (PFECAs) and used in company-specific processes.^10^ Examples include Chemours’ hexafluoropropylene oxide dimer acid (HFPO-DA, “Gen-X”), 3M Dyneon’s ammonium 4,8-dioxa-3*H*-perfluorononanoate (“ADONA”), the cyclic perfluorinated C6 quad-ether “C6O4”—used by Solvay/Syensqo, and perfluoro­(2-ethoxy-2-fluoroethoxy)-acetic acid (“EEA”) used by Asahi Glass Co. (AGC).^10,11,53^ In addition to the introduction of alternative substances, industry has also been pressured by authorities to introduce various emission abatement measures to limit emissions of fluorosurfactants and other PFAS.^12^ Although most replacement fluorosurfactants have a higher solubility and thus a lower bioaccumulation potential compared to PFOA and PFNA, their continued release, persistence, toxicity, and classification as PFAS warrant further research.^13^ Additionally, relatively little is known about the emissions of PFAS that are not used as fluorinated processing aids by the fluoropolymer production industry.

The aim of the current study was to investigate the emissions and occurrence of the replacement fluorinated processing aid EEA and other PFAS in air and water collected in the vicinity of an FPP operated by AGC Chemicals Europe in Thornton-Cleveleys, UK (Figure 1). Although a recently published study by Megson et al. also described PFAS suspects found in water close to this plant,^14^ this study is the first to quantify EEA in any matrix and one of the few investigating emissions by AGC Chemicals.

**Figure 1:**
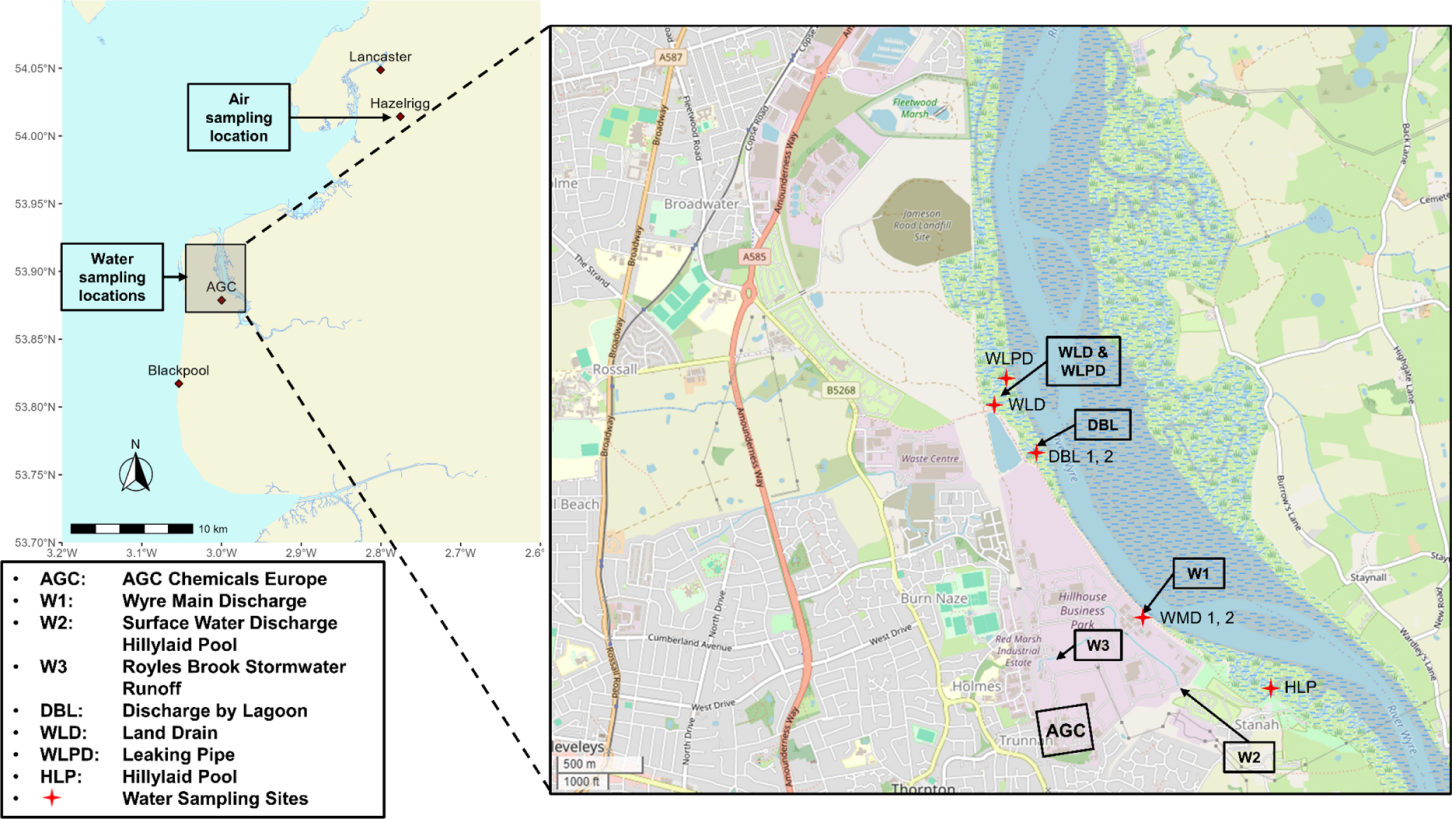
Map showing the locations of the fluoropolymer production plant of AGC Chemicals, discharges mentioned in the permit, water sampling locations, and the sampling location at the Hazelrigg Meteorological Station and the cities of Lancaster and Blackpool. The base map was obtained from OpenStreetMap contributors, available under the Open Database License (ODbL 1.0).^26^

## 2 Materials and Methods

### 2.1 Study Site

The AGC Chemicals Europe FPP produces both PTFE and (poly)­ethylene-tetrafluoroethylene (ETFE), with production volumes of 4000 and 2000 tonnes per year, respectively.^15^ For the production of PTFE, the plant uses emulsion polymerization, while ETFE is produced in granular form by using suspension polymerization. From 1955 to 2012, ammonium perfluorooctanoate (APFO) was used as a processing aid for PTFE production (Figure 4).^16^ The emissions of the FPP are regulated by the UK Environment Agency (UK EA) through an environmental permit.^15^ According to estimates by the UK EA, around 145 tonnes of PFOA, the dissociated form of APFO, were released to the local environment (75 tonnes to the River Wyre and 70 tonnes to the atmosphere) during this time. Additionally, <5 tonnes of PFOA were landfilled.^16^ From 2012 onward, APFO was replaced by EEA-NH_4_ (CAS 908020-52-0), the ammonium salt of a linear six-carbon PFECA with two ether linkages, one between the second and third carbon and one between the fourth and fifth carbon (Figure 2). Synonyms for this substance include SAA-1000, PFO2OA, and “Asahi’s product”.^10,13,15,17^ According to the UK EA, around 800 kg of the ammonium salt of EEA (EEA-NH_4_) is discharged into the River Wyre on a yearly basis.^13^ Additionally, <100 kg of the substance is emitted to the atmosphere.^13^ Neither AFPO nor its successor, EEA-NH_4_, has been produced on-site but instead has been imported. In addition to the fluoropolymers produced through polymerization, monomer tetrafluoroethylene (TFE) is also produced on-site through the pyrolysis of chlorodifluoromethane (HCFC-22).^15^

**Figure 2:**
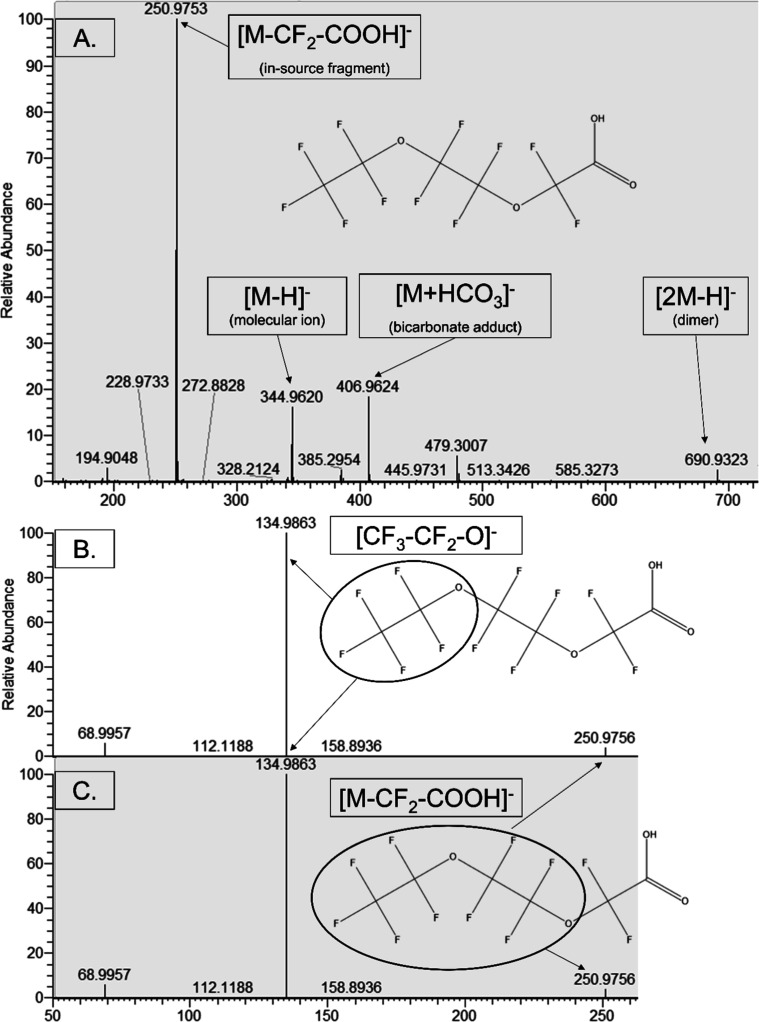
Typical full-scan mass spectrum of EEA (A) along with product ion scans of the major EEA in-source fragment (*m*/*z* 250.9753; B) and molecular ion (344.9620; C). Note the relatively low response of the molecular ion in the MS1 spectrum.

Emissions of HCFC-22, fluoroform (HFC-23), trifluoroethylene (TrFE), TFE, hexafluoropropylene (HFP), and difluoromethane (HFC-32) are permitted to the air, while small releases of perfluoroisobutylene (PFIB) are permitted to water.^6,15^ Additionally, emissions of 1H-perfluorohexane (1H-PFHx), perfluorobutyl ethylene (PFBE), and perfluoropropyl vinyl ether (PPVE) are permitted for which no reporting requirements are required.^6,18–20^

The plant has three discharge points to surface water that all ultimately lead to the River Wyre estuary (Figure 1). The main discharge point “W1” is a direct discharge to the River Wyre that, according to the emission permit, receives all process water effluent that has been treated by an on-site effluent treatment plant. The FPP shares this discharge point with the Victrex poly­(ether ether ketone) (PEEK) production plant that is located on the same industrial site. The second discharge point “W2” releases water to the Hillylaid Pool, a small tributary to the River Wyre. According to the environmental permit, only uncontaminated surface water and roof water from the installation are discharged here. The third discharge point, “W3”, is a storm runoff discharge that only discharges overflow from the west effluent pit under very high rainfall conditions to a local stream called Royles Brook.^15^

Atmospheric emissions of various PFAS and fluorinated organic substances were reduced through the installation of a thermal oxidizer facility in 2017, which treats both flue gases and liquid waste. In 2007, prior to the installation of this thermal oxidizer, PFOA levels of up to 828 pg/m^3^ were detected at the Hazelrigg Meteorological Station, one of the sampling sites of this study (Section 2.3.1).^21^ Additionally, part of the process water effluent is currently treated with a combination of techniques that aim to reduce PFAS emissions. These included ion exchange resins and activated carbon filters (personal communication with Stuart Ede of AGC Chemicals Europe).

### 2.2 Standards and Reagents

Standards for a total of 50 native PFAS (including EEA) as well as multiple isotope-labeled internal standards (ISs) and recovery standards (RSs) were obtained from Wellington Laboratories (Guelph, ON, CA) and Apollo Scientific (Bredbury, UK). A full list is provided in Table S1 of the Supporting Information.

### 2.3 Sampling and Extraction

#### 2.3.1 Air Samples

A total of 21 air samples were collected between June 11 and September 24, 2021 using a Tisch TE-1000 (Tisch Environmental, Cleves, OH, US) high volume air sampler located at the Hazelrigg Meteorological Station at Lancaster University on 110 mm quartz fiber filters (QFFs). This sampling site is located about 20 km northeast (downwind under prevailing conditions) of the AGC Chemicals Europe site in Thornton-Cleveleys (Figure 1) and was chosen because (a) PFAS measurements could be compared to a previous study^21^ from the same site; (b) meteorological data (e.g., wind speed and direction) are collected that could be analyzed as meteorological drivers of PFAS air concentrations; (c) it is located near-shore (6.5 km) and downwind of the FPP, giving the possibility of assessing the influence of sea-spray aerosol (SSA) on the atmospheric levels of PFAS; and (d) practical reasons, including a power supply for active sampling.

QFFs were baked in an oven at 450 °C for 24 h and subsequently packed in aluminum foil prior to use. A flow rate of 0.19–0.25 m^3^/min was used and about 1500 m^3^ of air was collected per sample, corresponding to sampling times between 96 and 165 h. All QFFs were extracted using ultrasonication in methanol, following previously described methods.^22,23^

#### 2.3.2 Surface Water Samples

Duplicate water samples were taken during a single sampling day (12-02-2023) at the main discharge of the site on the River Wyre (“W1”) and a discharge point by the lagoon north of the main discharge. Additionally, single water samples were taken at the surface water discharge near the Hillylaid Pool (the tributary receiving discharge “W2”), at a land drain to the north of the lagoon and a leaking pipe leading to the River Wyre close to this land drain (Figures 1 and S3–S7 in the Supporting Information). Unfortunately, the discharge point “W3” could not be sampled due to access limitations.

Water samples (50 mL each) were extracted in duplicate using Weak Anion Exchange (WAX) solid-phase extraction (SPE) cartridges (Waters, Milford, MA, USA) following previously described methods.^24^ Briefly, prior to extraction, the samples were spiked with 2000 pg ^13^C-labeled internal standards (ISs, Table S1 in the Supporting Information). The 3 cm^3^ (60 mg) WAX cartridges were conditioned using 4 mL of methanol (MeOH, Supelco LiChrosolv, Merck, Darmstadt, DE) with 0.1% ammonium hydroxide (NH_4_OH, 28–30% v/v, ACS-Reagent, Sigma-Aldrich, Steinheim, DE), and subsequently the cartridges were equilibrated using 4 mL of ultrapure water (Milli-Q, Merck, Darmstadt, DE). The samples were then loaded and washed with 2 mL of pH 4 acetate buffer containing 2 mM acetic acid (>99.8% ACS-Reagent, Sigma-Aldrich, Steinheim, DE) and 2 mM ammonium acetate (NH_4_CH_3_CO_2,_ ACS-Reagent, Merck, Darmstadt, DE) in Milli-Q and, subsequently, with 2 mL of MeOH. Elution was performed using 2 × 750 μL of MeOH with 0.1% NH_4_OH. After elution, the samples were evaporated to almost dryness and reconstituted with 130 μL of MeOH and 150 μL of Milli-Q with 4 mM of NH_4_CH_3_CO_2_, transferred to a LC–MS vial and spiked with 2000 pg Recovery Standard (RS) in 20 μL MeOH.

Additionally, due to the possibility of high PFAS concentrations expected in some samples, 300 μL aliquots were taken from all water samples for direct injection. Prior to analysis, the aliquots were spiked with 2000 pg of IS and passed through a nylon centrifuge filter in an Eppendorf tube. These were subsequently diluted 50:50 with MeOH and transferred to a 600 μL LC–MS vial, after which 2000 pg of RS was added, and the samples were ready for injection and analysis.

### 2.4 UHPLC-HRMS Analysis

Extracts were injected in a Dionex Ultimate 3000 ultrahigh-performance liquid chromatograph (UHPLC) linked to a Q-Exactive HF Orbitrap (Thermo Fisher Scientific, Waltham, MA, USA) high-resolution mass spectrometer (HRMS). The mobile phase consisted of A: Milli-Q: Acetonitrile (ACN, HPLC-grade, VWR, Rosny-sous-Bois-Cedex, FR) (95:5) and B: ACN: Milli-Q (99:1), both with 2 mM ammonium acetate (NH_4_CH_3_CO_2_) and was led through a Waters PFC Isolator column (Waters, Wilmslow, UK) after mixing. Subsequently, a Waters Acquity BEH C18 guard column (Waters, Wilmslow, UK) coupled with a 1.7 μm, 50 × 2.1 mm Waters Acquity BEH C18 column (both maintained at 50 °C) was used for separation. The analytes were ionized using an electrospray ionization source (ESI) operated in negative mode. The Orbitrap was used in full scan mode (scan range: 150–1800 Da, resolution: 120,000 fwhm) and data-dependent MS^2^ mode (scan range: 50-1800 Da, resolution: 15,000 fwhm), with an inclusion list created from around 5000 potential PFAS masses derived from homologous series of PF­(E)­CAs and PF­(E)­SAs with different moieties and in-source fragments.

### 2.5 Data Handling

The data analysis was conducted using Thermo Fisher TraceFinder version 4.1 software. Quantification of individual PFAS was based on relative response of exact masses (5 ppm mass error tolerance) to matched ISs using an 8-point calibration curve ranging from 0.03 to 135 pg/μL in solvent, corresponding to 0.18 and 810 ng/L in water, respectively, when accounting for the extraction ratio and assuming 100% recoveries. Because isotopically labeled standards were not available for all PFAS in this study, surrogate internal standards (i.e., non-exact matches) were used to quantify certain substances as detailed in Table S1. The surrogate ISs were chosen based on similarities in molecular structure, functional groups, retention time, and recovery. IS recoveries were calculated by comparing their response areas to those of the RSs (^13^C_8_ PFOA or ^13^C_8_ PFOS; Table S1).

For the water samples, the instrumental limits of detection (LODs) and quantification (LOQs) were calculated by taking the standard error of the residuals from the three lowest points on the calibration curve. This value was then divided by the slope of the calibration curve and multiplied by 10 to determine the LOQ and by 3.3 to calculate the LOD. For the air samples, due to matrix effects leading to overall lower and more variable IS responses, method LODs (MDLs) and LOQs (MQLs) were determined. This was achieved by dividing the LOD or LOQ response areas by the response area of the internal standard in the sample and using this ratio as input in the calibration curve and using the volume of sampled air to arrive at a MDL or MQL concentration, respectively.

### 2.6 Quality Control

#### 2.6.1 Air Samples

Multiple field blanks were taken by inserting a QFF into the sampler and following the same storage and transport procedure as for the sample filters. Lab blanks were included in every extraction by adding in triplicate unused baked QFFs to the extraction procedure. Additionally, a spike-and-recovery test was performed by fortifying a QFF with native analytes (2000 pg each) and following the same extraction procedure.

#### 2.6.2 Water Samples

Included with the water SPE and direct injection samples were triplicate lab blanks consisting of Milli-Q water, and for the SPE-method, a spike-and-recovery test was performed consisting of Milli-Q water, which was additionally spiked with a 1000 pg mix of native (^12^C) PFAS.

### 2.7 Data Processing

The suspect screening method used Thermo Fisher TraceFinder version 4.1 software in conjunction with the Thermo Fisher Xcalibur Qual Browser. The suspect list was based on the inclusion list used in the HRMS method (Section 2.4) and also containedhomologous series of perfluoroalkyl (ether) acids with various moieties, functional groups, and in-source fragments and about 5000 exact masses. Suspect peaks were inspected manually for MS^2^ and in-source fragmentation patterns, isotopic ratios, and retention times, which were compared to available literature and the spectral libraries mzCloud and MassBank.

Using the Schymanski scale,^25^ suspects were either assigned level (1) when matched with an acquired reference standard; (2a) when an MS^2^ scan and a library spectrum match were acquired; (2b) without library match but with acquired MS^2^ and diagnostic evidence supporting the identification, such as retention time relative to PFCAs, presence of homologous series and fragmentation patterns in MS^1^; or (3) similar diagnostic evidence as 2b but without acquired MS^2^.^25^

### 2.8 Tracer Ion Analysis and Meteorological Parameters

Using previously described methodology,^22,23^ analyses of the presence and concentrations of SSA tracer ions were carried out on punches of all QFFs. Average wind speed and direction during sampling were calculated using eqs S1–S5 in the Supporting Information

## 3 Results and Discussion

### 3.1 QA/QC

Similar to HFPO-DA and various other PFECAs, EEA shows very pronounced in-source fragmentation.^27^ As such, the most intense fragment is not the molecular ion ([M – H]^−^) but rather the [M – CF_2_ – COOH]^−^ fragment, which could have an intensity up to an order of magnitude higher than the molecular ion (Figure 2). Therefore, this in-source fragment is used in the quantification method along with the molecular ion by summing the response. The retention time of EEA on the C18 column is similar to that of perfluoroheptanoic acid (PFHpA), indicating that the insertion of ether linkages slightly increases the hydrophobicity of the perfluoroalkyl chain as EEA has the same number of perfluorinated carbons and the same headgroup as PFHxA. Spike and recovery tests with EEA for both the QFF and water extraction methods yielded a recovery of 96% ± 6% and 109% ± 5%, respectively, and was similar to other PFAS with comparable perfluoroalkyl chain lengths (Table S3 in the Supporting Information).

### 3.2 Water Samples

#### 3.2.1 Targeted Analysis

The sum of detected PFAS concentrations (ΣPFAS) in water samples varied by nearly 3 orders of magnitude between the sampling locations (30–22,542 ng/L; Table S4). Remarkably, the main discharge point (W1) of the process water effluent had the lowest PFAS concentrations compared to all the other sampling points. At the main discharge, ΣPFAS concentrations were 30 ng/L (main discharge in Wyre 1) and 33 ng/L (main discharge in Wyre 2), while the other sampling points all had PFAS levels within the thousand to tens of thousands of ng/L (Figure 3). Overall three different PFAS profiles were encountered near the plant. The highest PFAS concentrations were found to the north of the plant at a discharge near a lagoon (DBL) and at a land drain north of the lagoon (WLD) and a leaking part of a long discharge pipe (WLPD) leading to the River Wyre. PFOA was very prominent in these samples, with concentrations ranging from 891 ± 37 ng/L to 20,624 ± 556 ng/L. Furthermore, PFOA concentrations in all samples were positively associated with those of other PFCAs, such as perfluorobutanoic acid (PFBA), perfluoropentanoic acid (PFPeA), PFHxA, PFHpA, and PFNA, which were also present in relatively high levels. There was also evidence of a positive association of PFOA concentrations with additional C_8_ PFAS, such as hydrogen-substituted PFOA, chlorine-substituted PFOA, and ether-PFOA (Section 3.2.2 suspect screening). Concentrations of EEA in these samples ranged from 120.8 ± 0.3 to 205.5 ± 1.0 ng/L and were in a similar range as the levels of the C_4_–C_7_ PFCAs.

**Figure 3:**
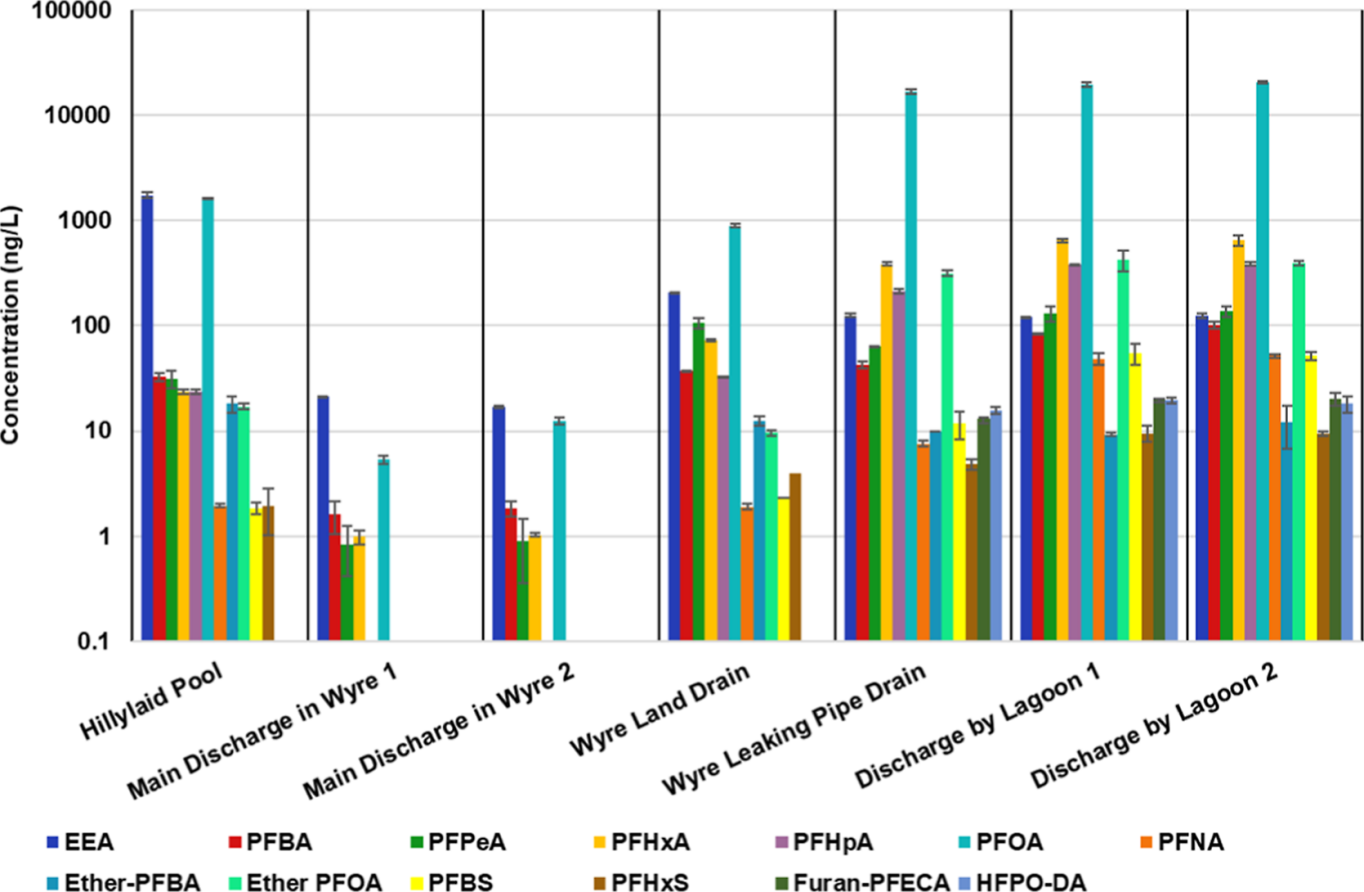
Levels of PFAS that were >LOD in the water samples taken around AGC Chemicals in Thornton-Cleveleys. Note the logarithmic scale.

The Hillylaid Pool water had the highest levels of the replacement fluorinated processing aid EEA out of all of the discharge locations (1744 ± 107 ng/L). However, in samples taken from the Hillylaid Pool and the main discharge, EEA had levels that were only slightly higher than those of PFOA (Figure 3). For duplicates collected at the main discharge, EEA concentrations were 21.17 ± 0.04 ng/L and 17.12 ± 0.40 ng/L, respectively, considerably lower than the concentrations at the Hillylaid Pool.

The levels of PFOA in the samples taken at the discharge by the lagoon (DBL) and the leaking pipe (WLPD) were quantified using direct injection, which also yielded similar concentrations and accuracy for PFOA as the SPE samples for the Hillylaid Pool and the Wyre land drain (Figure S1 in the Supporting Information). The PFOA concentrations of the SPE samples DLB and WLPD were not reported because the integrated areas of PFOA (maximum: ∼2 × 10^10^) fell well above the calibration range (maximum: ∼2 × 10^9^) and significant detector saturation effects occurred associated with these high PFOA concentrations, which led to suppression of the internal standard response and unreliable quantification (Table S4 in the Supporting Information).

The high PFOA concentrations are likely attributable to the extensive use of APFO for PTFE production at the plant between 1955 and 2012 and unabated emissions of PFOA to the environment during this time (Figure 4). Therefore, it could be that a large reservoir of PFOA and substances related to its production still exists within the perimeter of the industrial site. For instance, the samples with the highest PFAS levels were taken around a discharge by a lagoon to the north of the site. This lagoon was used as an effluent pit by Imperial Chemical Industries (ICI), the former operator of the PTFE production plant and was, unfortunately, not accessible during sampling. The high PFAS concentrations measured here indicate this lagoon should be the subject of further investigation. Furthermore, documentation of the UK EA points to the existence and former use of various other discharges, including a “North” discharge that might have been in use for a limited amount of time.^28^ Additionally, two landfills to the north of the site (Hillhouse and Jameson Road) were used until 2014 for disposal of material from the plant, which included <5 tons of PFOA.^16^ These landfills could potentially be leaching large amounts of PFAS into the environment.

**Figure 4:**
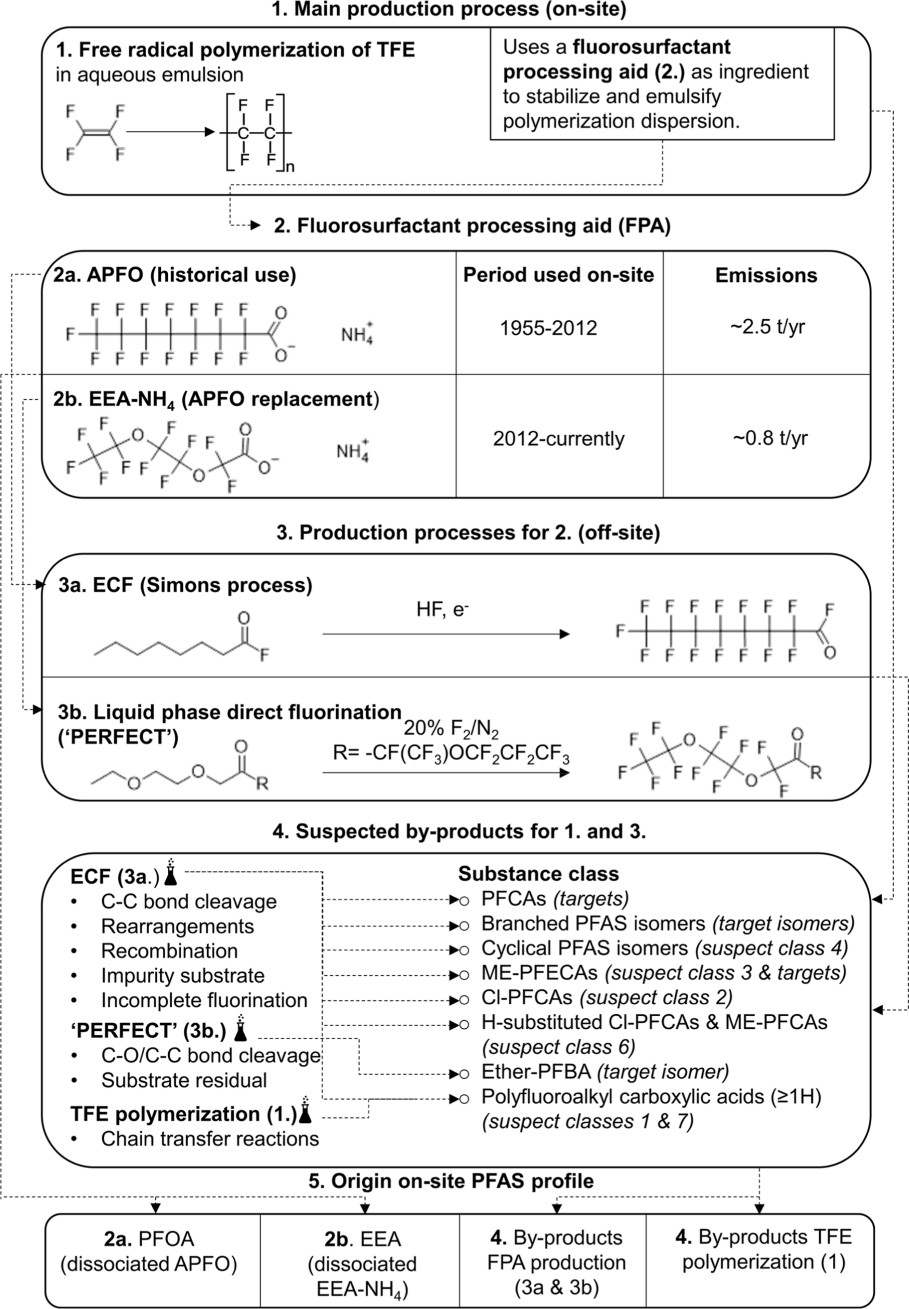
Overview of the substances and on-site and off-site processes that could contribute to the PFAS profile measured in the samples.

AGC Chemicals Europe has admitted that small amounts of PFOA and other PFCAs are formed nonintentionally due to a process in which the molecular weight of PTFE micropowders is altered using irradiation and thermal degradation.^29^ These impurities are removed from the polymer on-site, and whether subsequent emissions occur remains unclear. However, it does not seem likely that emissions could account for the levels found in the discharges, as a maximum PFOA concentration in the ppb range is expected in the PTFE polymer itself.^29^ It should be noted that the environmental permit of AGC Chemicals Europe did not mention the emissions of, e.g., PFOA or any other PFCAs; the only ionic PFAS that was covered by the permit is EEA-NH_4_.^15^

Another interesting aspect of the detected PFCAs is the prominence of branched isomers. These were identified by their shorter retention times and more pronounced in-source fragmentation (e.g., decarboxylation and lower relative [M – H]^−^ associated with shorter perfluoroalkyl chains) relative to the linear isomer and IS (Figure S8 in the Supporting Information). Both the presence of PFCAs of multiple chain lengths and the formation of branched isomers and certain PFAS suspect structures (Section 3.2.2) are associated with PFCA production through electrochemical fluorination (ECF) or the Simons process (Figure 4).^30^ This process is known to yield various structural isomers of PFOA as impurities.^31^ Although ECF production of APFO was carried out off-site, the linear isomer was not effectively isolated when used as a fluorinated processing aid; a mixture of homologues and isomers was used instead.^9^ Thus, these impurities were most likely released with PFOA after use of the polymerization process. Although the levels of branched isomers seem to be positively associated with PFOA levels, all samples were taken postpolymerization. Therefore, it could not be fully excluded that the formation of polymerization byproducts also plays a role in the presence of these substances.

Additional future research and sampling is necessary around the AGC Chemicals plant in order to verify the very high PFAS levels (thousands to tens of thousands of ng/L) and possible emissions from these “alternative” discharges. Elevated PFAS concentrations at these locations could potentially be explained by re-emission from existing on-site PFAS reservoirs. However, it is crucial to rule out the possibility that current production processes still involve either the use or the formation of significant quantities of PFOA and other PFAS, which could lead to subsequent emissions. Further investigation is also needed to determine whether process water discharge points other than W1 exist.

The high concentration of EEA (1744 ± 107 ng/L) found in the Hillylaid Pool is especially interesting in this regard. Due to this elevated level and the occurrence of the suspects of class 7 (Section 3.2.2), the PFAS profile in this water might more accurately reflect current use and emissions. Additionally, although there is a limited number of samples, only the concentrations of monoether-PFBA seem to be positively associated with those of EEA. This is opposed to the concentrations of all other detected targets, which were positively associated with PFOA. The detected monoether-PFBA was identified as an isomer of monoether-PFBA in the calibration standard. This isomer had the ether linkage between the second and the third carbon, instead of between the third and the fourth as shown by the C_2_F_5_O^–^ fragment present in its MS^2^ spectra instead of CF_3_O^–^ (Figure S15).

AGC currently uses liquid-phase direct fluorination instead of ECF or telomerization for producing EEA and other fluorochemical building blocks. This process (which takes place at a different AGC site) is described by Okazoe^32^ and involves the direct fluorination (using elemental F_2_) of a partially fluorinated ester substrate. The ester is synthesized through the reaction of a perfluorinated ether acyl fluoride and a nonfluorinated hydrocarbon alcohol. After fluorination, the ester is separated, and the original acyl fluoride is used to form a new partially fluorinated ester in another fluorination cycle, while the newly generated acyl fluoride is separated as the end product. Yields of this process are reportedly not 100% and, e.g., the cleavage of C–C bonds is described.^32^ Considering this, the fluorinated substrate used and because of the positive association with EEA concentrations, it could be that ether-PFBA is a byproduct of this direct fluorination process (Figure 4). Thus, it should be further investigated whether this method still leads to significant formation of additional impurities and byproducts and if these products could be present in the fluorinated processing aid EEA-NH_4_ that is used on-site.

Furthermore, it should be emphasized that PFAS concentrations found in these water samples are potentially problematic regardless of their origins and should be abated in order to prevent further contamination of the River Wyre and the estuarine area. PFOA concentrations alone are up to 4600 times higher than the proposed EU surface water limit (AA-EQS) of 4.4 ng/L.^33^ Additionally, concentrations reported here represent only one point in time. EEA concentrations in water at the main discharge point “W1” have been found to be substantially higher during a period in the second quarter of 2023 when the thermal treatment plant was offline. During this time, a peak EEA concentration of 12 mg/L (more than 500,000 times the concentration reported in this study; 21.17 ng/L) was reported to the UK EA.^34^

Comparison of measured PFAS levels with monitoring data from other fluoropolymer production plants in Europe shows that the highest PFAS concentrations reported in this study (∼μg/L range) are similar to levels found in process water discharges of other sites.^6,35,36^

#### 3.2.2 Suspect Screening

In addition to the PFAS that were quantified using standards, a total of 55 different PFAS suspects were identified in the water samples through the analysis of homologous series consisting of multiple per- or polyfluoroalkyl chain lengths (–CF_2_–)_*x*_ with values of “*x*” between 4 and 12 (Table S10 in the Supporting Information). These suspects were classified into seven groups: 1 hydrogen-substituted PFCAs (H-PFCAs), 2 chlorine-substituted PFCAs (Cl-PFCAs), 3 monoether PFECAs (ME-PFECAs), 4 cyclical monoether PFECAs (Cyc. ME-PFECAs), 5 dihydrogen-substituted PFOA (6:2 FTA and structural isomers), 6 hydrogen-substituted homologues of classes 2 and 3, and 7 polyhydrogenated PFCAs (H = 3–5).

The highest response for suspect classes 1–6 were found in the samples with the highest targeted PFAS levels taken at the discharge close to the lagoon (Table S9 in the Supporting Information). Furthermore, most of the detected homologous series had the highest response area at the C_8_ homologue (Table S9 and Figure S9–S12 in the Supporting Information). Response areas in the quantification injections of these samples for C_8_ homologues were comparable to those of EEA: H-PFOA (8.8 × 10^7^), Cl-PFOA (2.8 × 10^7^), cyclic-ether PFOA (2.8 × 10^7^), and EEA (4.9 × 10^7^). Thus, when assuming similar response factors for these suspects as PFOA, the concentrations of these suspects in this sample could be similar to those of EEA and C_4_–C_7_ PFCAs and in the hundreds of ng/L. Although the response areas were lower, these suspects were identified in other samples too (Table S8 in the Supporting Information) and have been identified in other studies close to fluoropolymer production plants in Asia.^37,38^ Because differences in response areas between samples cannot be directly interpreted as concentration differences (because of factors such as varying ionization efficiencies and recoveries), we used them for exploratory comparison purposes. For further validation, we also normalized the response areas of all suspects using the average response areas of C_4_–C_12_ PFCA ISs in the samples. This normalization approach demonstrated good consistency and comparability across samples (Table S11).

Because of the high responses of H-PFCA suspects in the samples taken at the discharge by the lagoon, these extracts were reinjected with acquired calibration standards of H-PFOA, H-PFNA, and H-PFUnDA. The reinjection confirmed the identity of these suspects, and quantification revealed H-PFOA concentrations up to 1027 ± 41 ng/L (Table 1). As only PFOA and EEA showed higher concentrations in this study, this further highlights the importance of applying high-resolution mass spectrometry approaches on samples influenced by FPP emissions.

**Table 1:** Concentrations of the H-PFCAs; H-PFOA (C_8_), H-PFNA (C_9_), and H-PFUnDA (C_11_) (ng/L) in the Water Samples Taken Close to the Lagoon near AGC Chemicals Following Reinjection with Acquired Calibration Standards

sample name	H-PFOA	H-PFNA	H-PFUnDA
discharge by lagoon 1	927 ± 21	62 ± 2.	11.1 ± 4.2
discharge by lagoon 2	1027 ± 41	63 ± 0.11	11.0 ± 1.3

For all suspect classes, it is probable that multiple branched isomers are present in the samples, due to multiple peaks for the same exact masses at earlier retention times in the chromatograms, similar to the PFCAs. Due to the very high concentrations of PFOA with a probable ECF signature in these samples (tens of thousands of ng/L) and the positive association of the response areas of most suspects with PFOA levels (Table S10), it is likely that most of these suspects are byproducts of ECF (Figure 4). Molar yields of linear PFOA from ECF were usually quite poor (15–20%).^39^ Various impurities are formed through incomplete fluorination, bond cleavages, rearrangements, and recombination and from possible impurities of the substrate.^39^ As such, substances such as polyfluoroalkyl carboxylic acids (e.g., suspect classes 1, 6, and 7) along with the cyclical monoether PFECAs have also been reported as impurities or byproducts of ECF.^31,40^ Additionally, Cl-PFCAs have also been reported in Asia, where ECF PFOA is still used.^37^ Octanoyl chloride is used as a starting material for the ECF substrate octanoyl fluoride.^9^ Reportedly, chlorine on a terminal carbon is effectively retained under ECF.^41^ Because rearrangements are common during this process, we hypothesize that Cl-PFCAs could potentially stem from impurities in the ECF substrate. Unfortunately, and similar to earlier reported data from Asia,^37,42^ it remains difficult to acquire accurate MS^2^ spectra for Cl-PFCAs using Orbitrap HRMS. Although MS^2^ scans are triggered by the molecular ions, these scans contain only noise and no clear fragment peaks. We suspect that only a chlorine fragment (*m*/*z* = 35 or 37) is ionized, which is below the lower mass threshold (*m*/*z* = 50) of the Orbitrap scan range and that the rest of the molecule could form a neutral loss. Still, and similar to PFCAs, decarboxylated fragments were detected for Cl-PFCAs in MS^1^ (Figure S9 in the Supporting Information). These fragments also triggered MS^2^ scans with issues similar to those of the molecular ion.

Other possible formation processes may also contribute to the presence of the reported suspects. Chain transfer reactions are unavoidable when polymerizing TFE and lead to the inherent formation of various low molecular weight oligomers as byproducts (Figure 4). H-PFCAs and CH_3_-capped PFCAs, for instance, are known polymerization byproducts in the production of PTFE.^8^ Additionally, environmental processes could also contribute to the formation of certain PFAS found in the samples. For example, H-PFCAs have been reported to form as a result of reductive dechlorination of Cl-PFCAs.^43^ Other well-known environmental formation processes include the environmental transformation of various polyfluorinated precursors into PFCAs.^44^

In addition to the highest EEA concentrations, samples from the Hillylaid Pool also had the highest response areas of class 7 suspects. Due to the multiple hydrogen substitutions, it could be that these suspects are more susceptible to biotransformation than PFAS with perfluoroalkyl chains and therefore over time have gradually disappeared from the other PFAS reservoirs. This observation, along with the relatively high levels of EEA and the overall different PFAS profile in the Hillylaid Pool compared to the samples taken north of the main discharge, could support the hypothesis that the PFAS profile measured in the Hillylaid Pool reflects recent emissions. Thus, although the class 7 suspects could be byproducts of ECF through incomplete fluorination, we suspect that these substances measured in the Hillylaid Pool mostly stem from polymerization residuals through chain transfer reactions. The PFAS profile of the samples taken north of the discharge, where ECF PFOA dominates, could reflect the leaching of on-site PFAS reservoirs that have accumulated over multiple years. Similar to targets described before (except EEA), the environmental permit of AGC Chemicals Europe does not cover the emissions any of the suspects described above.^15^

### 3.3 Air Samples

PFAS were detected in all air samples taken at the Hazelrigg Meteorological Station with concentrations ranging from 0.29 to 5.43 pg/m^3^ (Figure 5 and Table S5). PFOA was prominent in these samples, with detection in 20 of 21 of these samples and concentrations ranging from 0.51 to 2.50 pg/m^3^ (Figure 5). Branched PFOA isomers were also detected in all of these samples. For the one sample where PFOA was not detected, the response of the IS was very poor for unknown reasons, resulting in high MDLs/MQLs for all PFCAs (Tables S6 and S7 in the Supporting Information). High air concentrations of PFOA and ΣPFAS were associated with high wind speeds from the southwest direction (Figure 5 and Table S5). PFOS and PFHxS were detected in all air samples, albeit with lower peak concentrations (PFOS; 0.49 pg/m^3^) or at levels between MDL and MQL (PFHxS).

**Figure 5:**
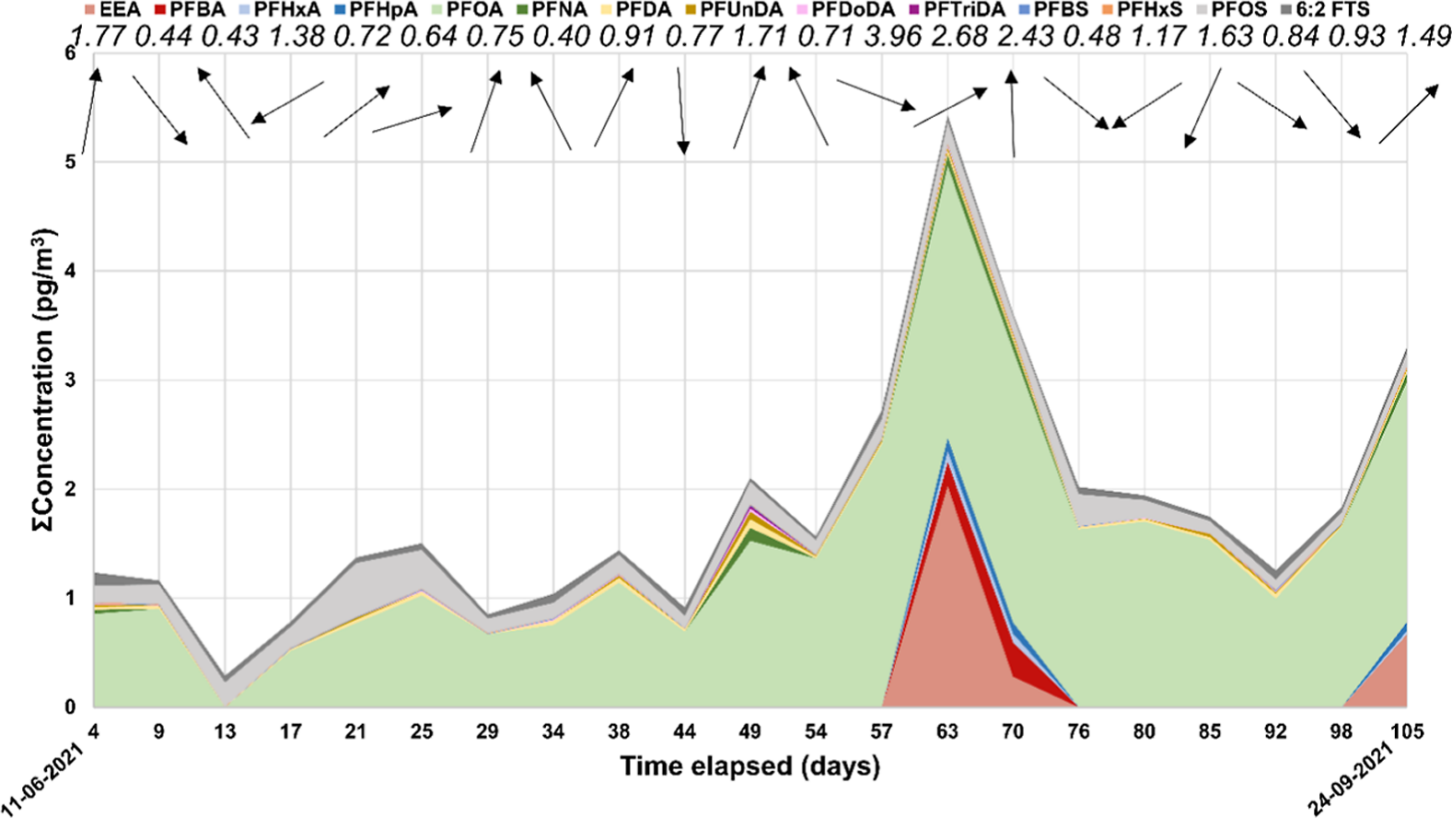
Air concentrations of PFAS that were >MDL at the Hazelrigg Meteorological Station during the sampling period. The arrows note the average wind direction during the sampling time, while the values above are the average wind speed (ms^–1^).

Notably, EEA was detected in only three of the four samples with the highest PFOA concentrations. The concentrations of EEA in these samples were 0.28 (between MDL and MQL), 0.66, and 2.02 pg/m^3^, and PFHxA and PFBA were also above MDL in these samples but below MQL, while PFHpA and the long-chain PFNA, PFDA, and PFUnDA were above MQL, and PFDoDA was above MQL in the two of these samples (Figures 5 and 6 and Table S5 in the Supporting Information).

**Figure 6:**
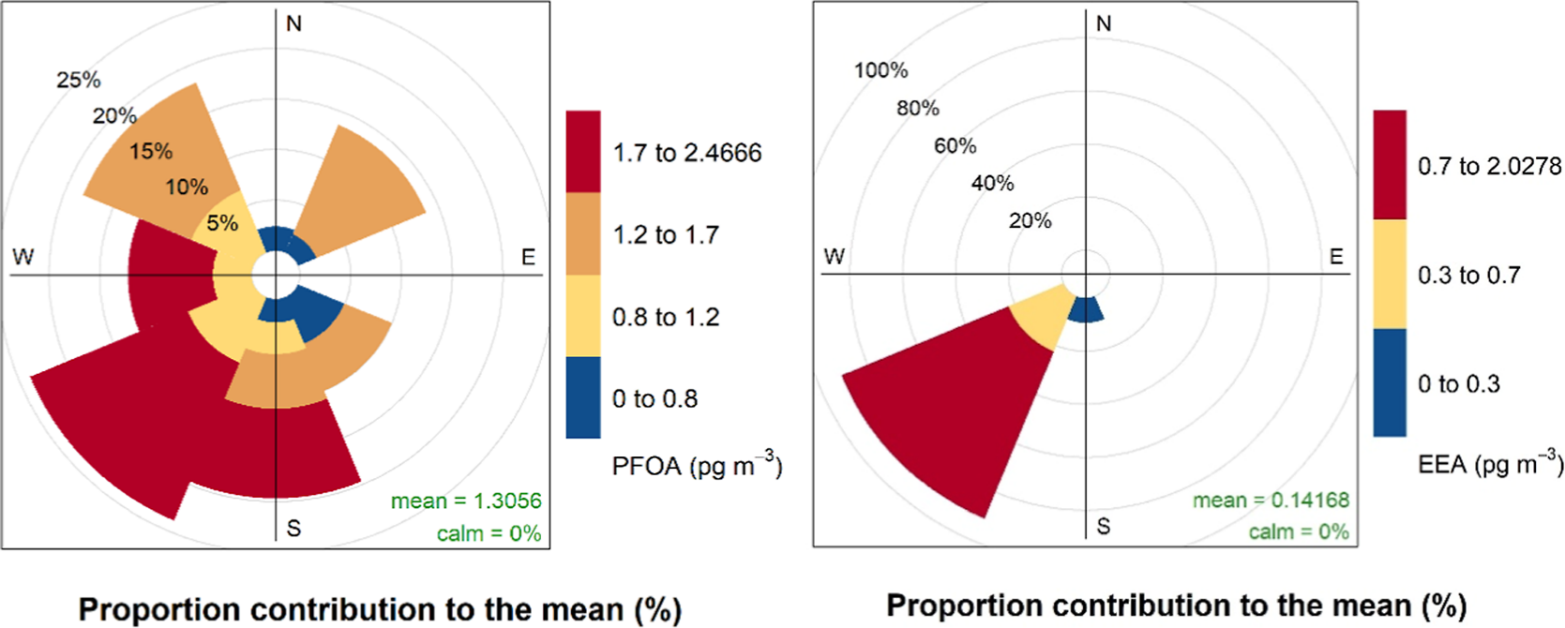
Pollution roses^51^ of PFOA (left) and EEA (right) at the Hazelrigg Meteorological Station, showing which wind directions contributed the most to mean PFOA and EEA air concentrations.

The presence of PFOA, EEA and the other PFCAs in the samples coincided not only with high wind speeds from the direction of the AGC Chemicals plant but also with high sodium (Na^+^) tracer ion concentrations that are a proxy for the levels of sea-spray aerosols (SSA). The two air samples (HAZ-14 and HAZ-15) with the highest PFOA concentrations, EEA responses, and only samples with C_9_–C_12_ PFCA levels all above MQL and with responses of the suspects previously described were also samples with highest Na^+^ levels (Table S5 in the Supporting Information). For PFOA and PFHxS, the Spearman’s rank correlation coefficients (ρ) with Na^+^ were 0.41 and 0.50, respectively. However, for PFOA, this correlation was not statistically significant by a slight margin (*p* = 0.07), while for PFHxS, it was (*p* = 0.02). As such, the concentrations of atmospheric PFOA could also be influenced to a minor extent by other sources, such as direct emissions by AGC Chemicals, the resuspension of previously deposited PFOA from other areas, and transformation of volatile precursors or urban emissions. PFOS did not show a statistically significant correlation with Na^+^ (ρ = 0.17, *p* = 0.45), and for the other analytes, the concentrations and detection frequencies were too low to draw conclusions on correlations with the Na^+^ tracer ion.

In the samples with the highest PFOA and EEA concentrations, trace responses of some of the suspects described in Section 3.2.2 were also detected (Figures S12 and S13 in the Supporting Information). Compared to the water samples, these responses and those of the other PFCAs were more pronounced for the longer-chained homologues (>C_8_). SSA enrichment factors of perfluoroalkyl acids with a perfluoroalkyl chain length of seven or more are significantly higher than those with shorter perfluoroalkyl chain lengths.^45,46^ As such, with similar water concentrations, air concentrations of these homologues would be relatively higher compared to their shorter-chained counterparts. On the other hand, short-chain PFAS have similar or higher long-range atmospheric transport potential compared to long-chain PFAS. This is because these PFAS preferentially bind to particles in the accumulation mode (diameter 0.1–1.0 μm), which remain suspended in the atmosphere for longer periods than other particle sizes^47,48^ If direct emissions were the dominant source for the measured air PFAS concentrations, the relative concentrations of short-chained homologues and PFCAs would be expected to be similar at Hazelrigg. However, these are measured at lower concentrations or not measured at all compared to concentrations in the water in the direct vicinity of the plant. More sampling data needs to be collected to statistically strengthen the correlations between air concentrations of PFOA, EEA, long-chain PFAS, and the Na^+^ tracer ion at Hazelrigg. Although the current evidence remains largely circumstantial and based on a limited sample size (*n* = 21), it is suspected that enrichment of PFAS in SSA is currently the dominant driver of the atmospheric concentrations of PFAS related to AGC emissions measured in Hazelrigg. Additionally, the measured concentrations of PFOA (peak 2.50 pg/m^3^) and EEA (peak 2.02 pg/m^3^) are much lower than the PFOA peak level of 828 pg/m^3^ found in 2007,^21^ before the installation of the emission abatement system that could have significantly reduced the impact of air emissions directly from the stacks of the plant. However, documents from the UK EA indicate that this system was not operational during the entire first quarter of 2023 and part of the first quarter of 2024.^49,50^ Further investigation is necessary in order to understand how periods of nonoperational emission abatement affect environmental PFAS levels.

### 3.4 Limitations

It has to be stressed that the chemical space covered in this study was limited by (a) the sampling methods (high volume air sampling of PM10 particulates and grab sampling of surface water); (b) the extraction protocol (ultrasonication in MeOH for QFFs and WAX-SPE for surface water); and (c) instrumental analysis (chromatography using a C18 stationary phase and mass spectrometry using ESI^–^ ionization). As such, this study mainly focused on ionic PFAS ≥C_4_ and ≤C_18_ in surface water and airborne particles. Because of these limitations, additional PFAS were potentially emitted by AGC Chemicals that are not well covered by our methodology and thus not described in this study. For instance, AGC chemicals are reportedly emitting large amounts of volatile neutral PFAS to air, such as the fluorinated solvent 1H-PFHx (>30 t/yr) and the comonomer PFBE (900 kg/yr), both used in ETFE production.^18,19^ These emissions, unfortunately, could not be measured using our methods. Therefore, future studies should focus on expanding the chemical space of PFAS measurements around AGC Chemicals Europe and other plants in order to gain more integral insight into PFAS emissions from fluoropolymer production. Furthermore, the sampling times were limited to 1 day for the water samples and 105 days for the air sampling campaign. Especially for the water samples, additional sampling would capture more possible temporal variability in the PFAS concentrations.

### 3.5 Implications

This study shows that the AGC Chemicals site continues to be a source of legacy and scarcely monitored PFAS to the local environment through direct emissions and/or leaching of PFAS reservoirs such as old discharges, the local landfill, or lagoon. A large fraction of these emissions flow into the River Wyre estuary, which drains into the strongly tidal Morecambe Bay. This area is important as a wildlife habitat and for shellfish harvesting. Recently, elevated levels of PFOA were reported in otters with their habitats close to the AGC Chemicals plant.^52^ Further monitoring data are required to establish the extent of PFAS contamination on-site and in the River Wyre and Morecambe Bay food webs in order to assess the effects of the continued emissions of legacy and alternative PFAS from AGC Chemicals Europe. Very little is known about the properties of the scarcely monitored PFAS identified in this study, but one might assume given their similar structures to PFCAs that they would have similar persistence, bioaccumulation potential, and toxicities. Further research on the properties, including toxicities, of the identified PFAS is required before human and environmental risk assessments can be performed. Secondary emissions of PFAS from the ocean due to their enrichment on sea spray aerosol could lead to additional deposition of long-chain homologues in coastal areas around Morecambe Bay, and our study shows that sea spray aerosol transport of PFAS could also be an additional secondary source to air for several legacy PFAS (notably PFHxS, PFOA, and some long-chain PFCAs) as well as the replacement substance EEA and the scarcely monitored suspects (H-PFCAs, Cl-PFCAs, and ME-PFECAs). These identified suspects are most likely to be byproducts of electrochemical or liquid-phase fluorination or polymerization.^53,54^

## Supplementary Material



## Notes

Target analyte list, parameters of the instrumental method, calculations, sample concentrations, mass spectra, chromatograms, and photos of the sampling site (PDF)
