# Supplementary material for: Perfluoro(2-ethoxy-2-fluoroethoxy)-acetic Acid and Other Target and Suspect PFAS in the Vicinity of a Fluoropolymer Production Plant
Source: Environ Sci Technol. 2025 Jul 18;59(29):15420–31. doi: 10.1021/acs.est.5c07856 (PMC12312159; doi:10.1021/acs.est.5c07856)
Supplement: Supplementary file 1 [file es5c07856_si_001.pdf]

Supporting information for ‘Perfluoro(2-ethoxy-2-fluoroethoxy)-acetic acid (EEA) and other target and suspect PFAS in the vicinity of a fluoropolymer production plant’

Joost Dalmijn[a]*, Jonathan P. Benskin[a], Matthew E. Salter[a], Andrew J. Sweetman[b], Crispin J. Halsall[b] Jack Garnett[b] and Ian T. Cousins[a]*

[a] Department of Environmental Science, Stockholm University, SE-10691 Stockholm, Sweden
[b] Lancaster Environment Centre, Lancaster University, LA1 4YQ, Lancaster, United Kingdom

*Joost.Dalmijn@aces.su.se
*Ian.Cousins@aces.su.se

Summary: 39 pages, 15 figures, 12 tables.

Table S1. Analyte list of targets quantified in this study. Recovery standards are in ***bold****, italics and marked by an asterisk *. Instrumental limits of detection (IDL) and quantification (IQL) in pg/µL used for the water samples and air sampling method detection and quantification limits (MDL and MQL, pg/m³) based on average IS response in the samples.*

Name	Formula	Internal standard	CAS	m/z used	IDL	IQL	MDL	MQL
**Perfluoroalkyl carboxylic acids (PFCAs)**								
Perfluorobutanoic acid (PFBA)	$C_4HF_7O_2$	$^{13}C_4$ PFBA	375-22-4	212.9792	0.02	0.06	2.270	6.815
Perfluoropentanoic acid (PFPeA)	$C_4HF_9O_2$	$^{13}C_5$ PFPeA	2706-90-3	262.9760	0.16	0.50	0.022	0.100
Perfluorohexanoic acid (PFHxA)	$C_6HF_{11}O_2$	$^{13}C_2$ PFHxA	307-24-4	312.9728	0.04	0.11	0.006	0.216
Perfluorocyclohexane carboxylic acid (PFCHCA)	$C_7HF_{11}O_2$	$^{13}C_2$ PFHxA	374-88-9	324.9729	186.80	566.06	826	2504
Perfluoroheptanoic acid (PFHpA)	$C_7HF_{13}O_2$	$^{13}C_4$ PFHpA	375-85-9	362.9696	0.05	0.16	0.049	0.116
Perfluorooctanoic acid (PFOA)	$C_8HF_{15}O_2$	$^{13}C_4$ PFOA<br>***$^{13}C_8$ PFOA****	335-67-1	412.9666	0.09	0.28	0.001	0.039
Perfluorononanoic acid (PFNA)	$C_9HF_{17}O_2$	$^{13}C_5$ PFNA	375-95-1	462.9632	0.03	0.08	0.033	0.069
Perfluorodecanoic acid (PFDA)	$C_{10}HF_{19}O_2$	$^{13}C_5$ PFDA	335-76-2	512.9600	0.04	0.12	0.009	0.030
Perfluoroundecanoic acid (PFUnDA)	$C_{11}HF_{21}O_2$	$^{13}C_2$ PFUnDA	2058-94-8	562.9568	0.03	0.09	0.002	0.007
Perfluorododecanoic acid (PFDoDA)	$C_{12}HF_{23}O_2$	$^{13}C_2$ PFDoDA	307-55-1	612.9536	0.03	0.09	0.005	0.012

Perfluorotridecanoic acid (PFTriDA)	$C_{13}HF_{25}O_2$	$^{13}C_2$ PFDoDA	72629-94-8	662.9504	0.03	0.09	0.007	0.033
Perfluorotetradecanoic acid (PFTeDA)	$C_{14}HF_{27}O_2$	$^{13}C_2$ PFDoDA	376-06-7	712.9472	0.03	0.09	0.010	0.032
Perfluorohexadecanoic acid (PFHxDA)	$C_{16}HF_{31}O_2$	$^{13}C_2$ PFDoDA	67905-19-5	812.9408	0.08	0.27	0.007	0.039
Perfluorooctadecanoic acid (PFOcDA)	$C_{18}HF_{35}O_2$	$^{13}C_2$ PFDoDA	16517-11-6	912.9345	0.11	0.34	0.286	0.856
**Perfluoroalkane sulfonic acids (PFSAs)**								
Perfluorobutane sulfonic acid (PFBS)	$C_4HF_9O_3S$	$^{18}O_2$ PFHxS	375-73-5	298.9429	0.02	0.07	0.087	0.088
Perfluorohexane sulfonic acid (PFHxS)	$C_6HF_{13}O_3S$	$^{18}O_2$ PFHxS	355-46-4	398.9366	0.004	0.013	0.006	0.036
Perfluorooctane sulfonic acid (PFOS)	$C_8HF_{17}O_3S$	$^{13}C_4$ PFOS<br>***$^{13}C_8$ PFOS****	1763-23-1	498.9302	0.01	0.02	0.001	0.039
Perfluorodecane sulfonic acid (PFDS)	$C_{10}HF_{21}O_3S$	$^{13}C_4$ PFOS	335-77-3	598.9238	0.06	0.19	0.007	0.012
**Perfluoroalkylether carboxylic acids (PFECAs)**								
Perfluoro-3-methoxymethanoic acid (PMPA, ether-PFBA)	$C_4HF_7O_3$	$^{13}C_5$ PFPeA	377-73-1	228.9738	1.15	3.48	29.8	90.1
Perfluoro-4-methoxybutanoic acid (PMBA, ether-PFPeA)	$C_5HF_9O_3$	$^{13}C_5$ PFPeA	863090-89-5	278.9709	0.01	0.04	0.225	0.619
Perfluoro-3-tetrahydro-2-furancarboxylic acid (Furan-PFECA)	$C_5HF_7O_3$	$^{13}C_5$ PFPeA	65578-62-3	196.9836	0.30	0.91	75.5	229
Perfluoro-3,6-dioxaheptanoic acid (PFO2HpA)	$C_5HF_9O_5$	$^{13}C_2$ PFHxA	151772-58-6	200.9787	0.01	0.04	0.201	0.518
Hexafluoropropylene oxide dimer acid (HFPO-DA)	$C_6HF_{11}O_3$	$^{13}C_3$ HFPO-DA	13252-13-6	284.9780	0.41	1.25	3.095	8.66
Perfluoro-4-isopropoxybutanoic acid (PFIPBA)	$C_7HF_{13}O_3$	$^{13}C_4$ PFOA	801212-59-9	378.9642	0.01	0.04	0.098	0.176
Perfluoro-3,6-dioxaoctanoic acid (PFO2OA, EEA)	$C_6HF_{11}O_4$	$^{13}C_4$ PFHpA	80153-82-8	250.9760	0.73	2.23	0.009	0.249
3H-Perfluoro-3-[(3-methoxy-propoxy)propanoic acid (DONA)	$C_7H_2F_{12}O_3$	$^{13}C_4$ PFHpA	919005-14-4	376.9689	0.01	0.04	0.031	0.067

8-Iodoperfluoro(6-oxaoctanoic) acid (IPFOOA)	$C_7HF_{12}IO_3$	$^{13}C_4$ PFOA	948014-44-6	486.8701	0.03	0.10	0.055	0.141
Perfluoro-3,6-dioxadecanoic acid (PFO2DA)	$C_8HF_{15}O_4$	$^{13}C_4$ PFOA	137780-69-9	350.9691	0.02	0.05	0.081	0.147
2-Perfluoropentoxy-2,3,3,3-tetrafluoropropanoic acid (PFPeOPA, ether-PFOA)	$C_8HF_{15}O_3$	$^{13}C_4$ PFOA	504435-11-4	428.9608	0.83	2.51	0.329	0.967
Perfluoro-3,6,9-trioxadecanoic acid (PFO3DA)	$C_7HF_{13}O_5$	$^{13}C_4$ PFOA	151772-59-7	316.9674	0.01	0.03	0.057	0.129
Hexafluoropropylene oxide trimer acid (HFPO-TA)	$C_9HF_{17}O_4$	$^{13}C_5$ PFNA	13252-14-7	184.9834	0.15	0.46	0.151	0.415
Perfluoro-3,6,9-trioxatridecanoic acid (PFO3TriDA)	$C_{10}HF_{19}O_5$	$^{13}C_5$ PFDA	330562-41-9	466.9575	0.01	0.04	0.025	0.048
Hexafluoropropylene oxide tetramer acid (HFPO-TeA)	$C_{12}HF_{23}O_5$	$^{13}C_2$ PFDoDA	65294-16-8	350.9691	0.01	0.03	0.038	0.079
Hexafluoropropylene oxide pentamer acid (HFPO-PeA)	$C_{15}HF_{29}O_6$	$^{13}C_2$ PFDoDA	65150-95-0	516.9550	0.02	0.07	0.492	1.471
Hexafluoropropylene oxide hexamer acid (HFPO-HxA)	$C_{18}HF_{35}O_7$	$^{13}C_2$ PFDoDA	52481-85-3	682.9400	0.11	0.32	0.248	0.710
**Fluorotelomer sulfonic acids (FTSAs)**								
4:2 fluorotelomer sulfonic acid (4:2 FTSA)	$C_6H_5F_9O_3S$	$^{13}C_2$ 6:2 FTSA	75746-90-8	326.9743	0.05	0.15	0.053	0.167
6:2 fluorotelomer sulfonic acid (6:2 FTSA)	$C_8H_5F_{13}O_3S$	$^{13}C_2$ 6:2 FTSA	27619-97-2	426.9679	0.01	0.03	0.016	0.044
8:2 fluorotelomer sulfonic acid (8:2 FTSA)	$C_{10}H_5F_{17}O_3S$	$^{13}C_2$ 6:2 FTSA	251099-16-8	526.9615	0.02	0.04	0.045	0.117
**Fluoroalkanesulfonamides (FASAs)**								
Perfluorooctanesulfonamide (FOSA)	$C_8H_2F_{17}NO_2S$	$^{13}C_8$ FOSA	754-91-6	497.9462	1.84	5.57	710	2153
N-ethylperfluorooctanesulfonamide (N-EtFOSA)	$C_{10}H_6F_{17}NO_2S$	d5-N-EtFOSA	4151-50-2	525.9775	0.01	0.03	-	-
N-methylperfluorooctanesulfonamide (N-MeFOSA)	$C_9H_4F_{17}NO_2S$	d3-MeFOSA	865-71-4	511.9618	0.01	0.04	-	-

Perfluorooctanesulfonamido acetic acid (FOSAA)	$C_{10}H_4F_{17}NO_4S$	d3-MeFOSAA	2806-24-8	555.9517	0.02	0.05	0.004	0.027
N-ethylperfluorooctanesulfonamido acetic acid (N-EtFOSAA	$C_{12}H_8F_{17}NO_4S$	D5-N-EtFOSAA	1336-61-4	583.9830	0.01	0.03	0.051	0.133
N-methylperfluorooctanesulfonamido acetic acid (N-MeFOSAA)	$C_{11}H_6F_{17}NO_4S$	d3-MeFOSAA	2355-31-9	569.9673	0.01	0.04	0.031	0.067
N-ethylperfluorooctanesulfonamidoethanol (N-EtFOSE)	$C_{12}H_{10}F_{17}NO_3S$	d5-N-EtFOSA	1691-99-2	630.0248	0.04	0.11	-	-
N-methylperfluorooctanesulfonamido ethanol (N-MeFOSE)	$C_{11}H_8F_{17}NO_3S$	d5-N-EtFOSA	24448-09-7	616.0092	0.11	0.33	-	-
**Chlorinated perfluoroalkylether sulfonic acids (Cl-PFESAs)**								
2-(6-chloro-dodecafluorohexyloxy)-tetrafluoroethane sulfonic acid (F53-B Major)	$C_8HClF_{16}O4_S$	$^{13}C_5$ PFDA	756426-58-1	530.8956	0.01	0.04	0.001	0.003
11-Chloroeicosafluoro-3-oxaundecane-1-sulfonic acid (F53-B Minor)	$C_{10}HClF_{20}O_4S$	$^{13}C_5$ PFDA	83329-89-9	630.8892	0.03	0.10	0.004	0.008
**Hydrogen-substituted perfluoroalkyl carboxylic acids (H-PFCAs)***								
H-perfluorooctanoic acid (H-PFOA)	$C_8H_2F_{14}O_2$	$^{13}C_4$ PFOA	13973-14-3	394.9758	0.04	0.13	-	-
H-perfluorononanoic acid (H-PFNA)	$C_9H_2F_{16}O_2$	$^{13}C_5$ PFNA	76-21-1	444.9726	0.05	0.14	-	-
H-Perfluoroundecanoic acid (H-PFUnDA)	$C_{11}H_2F_{20}O_2$	$^{13}C_2$ PFUnDA	1765-48-6	544.9663	0.04	0.13	-	-

*Analyzed at a later stage using a separate calibration curve

Table S2. Orbitrap source parameters, scan settings and LC-gradient

HESI settings			
Spray voltage	3.7 kV		
Capillary temperature	320 °C		
Sheath gas	30 arbitrary units (au)		
Aux gas	10 au		
Aux gas heater temperature	320 °C		
HRMS settings			
AGC target	Full scan: 3 000 000, ddMS2: 500 000		
Resolution	Full scan: 120 000, ddMS2: 15 000 FWHM		
HCD	35		
Mobile phase gradient			
Time (min)	Mobile phase A (%)	Mobile phase B (%)	Flow (mL/min)
0.0-0.5	90	10	0.4
0.5-8.0	90>1	10>99	0.4
8.0-11.0	1	99	0.4
11.0-11.1	1>90	99>10	0.4
11.1-13	90	10	0.4

Table S3. Results of the spike recovery tests (%) for both the quartz fiber filters as the WAX-SPE extraction methods

Substance	QFF-1	QFF-2	QFF-3	Average	STD	SPE-1	SPE-2	SPE-3	Average2	STD3
**PFBA**	100.59	94.10	94.30	96.33	3.69	94.60	93.70	94.10	94.13	0.45
**PFPeA**	93.99	87.43	89.62	90.35	3.34	91.76	91.64	96.39	93.26	2.71
**PFHxA**	112.20	106.87	106.05	108.38	3.34	90.70	95.00	92.90	92.87	2.15
**PFHpA**	103.80	102.38	100.01	102.06	1.91	88.09	91.37	88.19	89.22	1.87
**PFOA**	109.33	109.20	107.69	108.74	0.91	92.20	95.00	94.00	93.73	1.42
**PFNA**	112.00	109.81	107.22	109.68	2.39	85.30	85.20	86.10	85.53	0.49
**PFDA**	123.21	116.99	114.46	118.22	4.50	91.60	100.10	98.20	96.63	4.46
**PFUnDA**	116.44	110.36	106.46	111.09	5.03	116.60	120.50	121.10	119.40	2.44
**PFDoDA**	125.53	121.63	116.37	121.18	4.60	85.50	88.00	91.00	88.17	2.75
**PFBS**	107.70	101.80	99.63	103.04	4.18	107.16	94.43	95.11	98.90	7.16
**PFHxS**	108.61	106.50	103.88	106.33	2.37	98.94	96.91	97.34	97.73	1.07
**PFOS**	126.91	121.29	124.35	124.19	2.82	103.85	106.35	107.40	105.87	1.82
**6:2 FTS**	117.22	115.76	112.13	115.04	2.62	102.41	95.81	100.32	99.51	3.37
**EEA**	133.10	121.46	135.74	130.10	7.60	109.34	116.43	119.76	115.18	5.32
**Ether-PFBA**						102.27	93.34	103.28	99.63	5.47
**Ether-PFOA**						68.90	58.80	61.50	63.07	5.23
**PFCHCA**						73.40	84.30	76.00	77.90	5.69
**Ether-PFPeA**						106.67	100.91	106.79	104.79	3.36
**Furan-PFECA**						104.64	98.42	105.32	102.79	3.80
**PFO2HpA**						87.60	83.70	86.80	86.03	2.06
**HFPO-DA**	94.47	94.86	91.86	93.73	1.63	104.88	105.40	103.43	104.57	1.02
**PFIPBA**						110.13	110.88	113.10	111.37	1.54
**DONA**	94.60	90.75	87.24	90.86	3.68	99.35	101.43	103.82	101.53	2.24
**IPFOOA**						84.50	70.40	85.30	80.07	8.38
**PFO2DA**						72.60	76.40	69.80	72.93	3.31

**PFO3DA**						92.30	96.50	92.20	93.67	2.45
**HFPO-TA**						83.00	78.90	77.80	79.90	2.74
**PFO3TriDA**						81.70	99.70	102.40	94.60	11.25
**HFPO-TeA**						47.30	36.80	38.60	40.90	5.62
**HFPO-PeA**						14.00	3.00	3.00	6.67	6.35
**HFPO-HxA**						16.00	4.10	7.30	9.13	6.16
**4:2 FTSA**	115.37	109.16	110.22	111.58	3.32	95.81	101.97	105.16	100.98	4.75
**8:2 FTSA**	101.24	106.59	113.12	106.99	5.95	232.43	150.15	182.60	188.39	41.44
**FOSA**	90.92	83.63	84.48	86.34	3.98	431.36	678.93	418.87	509.72	146.67
**N-EtFOSA**	100.91	98.89	93.37	97.73	3.90	69.24	165.95	246.73	160.64	88.87
**N-MeFOSA**	183.71	0.00	87.21	90.31	91.89	68.36	112.04	149.42	109.94	40.57
**FOSAA**	76.40	91.15	88.84	85.47	7.93	98.38	46.18	52.21	65.59	28.56
**N-EtFOSAA**	101.93	99.76	94.32	98.67	3.92	80.94	80.94	82.31	81.40	0.79
**N-MeFOSAA**	114.40	111.61	119.09	115.03	3.78	81.32	81.76	83.46	82.18	1.13
**N-EtFOSE**	14.41	33.59	5.45	17.82	14.38	79.41	137.21	626.16	280.93	300.37
**N-MeFOSE**	10.34	0.00	2.53	4.29	5.39	80.96	317.00	587.79	328.58	253.62
**F53-B Major**	97.51	97.44	95.19	96.71	1.32	79.36	87.19	95.96	87.50	8.31
**F53-B Minor**	91.16	94.23	94.44	93.28	1.84	63.60	43.10	49.90	52.20	10.44

*Table S4. Concentrations of PFAS (ng/L) and limits of detection and quantification (ng/L) in water samples taken near AGC Chemicals Europe. Values in **bold** determined by direct injection.*

Substance<br>Sample Name	EEA	PFBA	PFPeA	PFHxA	PFHpA	PFOA	PFNA	Ether PFBA	HFPO-DA	Ether PFOA	Furan-PFECA	PFBS	PFHxS	ΣSum
**Hillylaid Pool**	1744.1 ±106.79	32.95 ±2.96	31.35 ±5.98	23.9 ±1.27	23.67 ±1.40	1624.6 ±30.25	1.96 ±0.08	18.12 ±3.08	<LOD	17.16 ±1.05	<LOD	1.86 ±0.25	1.92 ±0.91	*3521.59*
**Main Discharge in Wyre 1**	21.17 ±0.04	1.6 ±0.54	*0.84* ±0.42	0.99 ±0.16	<LOD	5.34 ±0.42	<LOD	<LOD	<LOD	<LOD	<LOD	<LOD	<LOD	*29.94*
**Main Discharge in Wyre 2**	17.12 ±0.40	1.84 ±0.28	*0.91* ±0.55	1.03 ±0.04	<LOD	12.44 ±0.99	<LOD	<LOD	<LOD	<LOD	<LOD	<LOD	<LOD	*33.34*
**Wyre Land Drain**	205.51 ±0.98	37.63 ±0.01	105.84 ±11.60	73.02 ±0.11	32.84 ±0.11	891.42 ±37.08	1.90 ±0.11	12.57 ±1.29	<LOD	9.63 ±0.63	<LOD	2.32	3.92	*1376.60*
**Wyre Leaking Pipe Drain**	124.65 ±4.99	42.9 ±3.17	64.36 ±0.08	386.16 ±13.07	212.48 ±13.21	**16588.89 ±784.47**	7.56 ±0.45	9.94 ±0.00	15.72 ±1.33	314.55 ±17.64	13.11 ±0.44	11.75 ±3.38	4.84 ±0.59	*17796.91*
**Discharge by Lagoon 1**	120.83 ±0.27	83.48 ±0.68	131.01 ±23.01	650.22 ±25.54	379.05 ±0.86	**19586 ±1051.33**	48.54 ±5.97	9.28 ±0.37	19.58 ±1.05	427.16 ±95.54	19.63 ±0.43	54.41 ±12.37	9.53 ±1.63	*21539.39*
**Discharge by Lagoon 2**	123.92 ±7.24	101.14 ±8.00	136.26 ±14.96	653.1 ±71.45	383.66 ±14.26	**20624.44 ±556.28**	51.56 ±1.47	12.07 ±5.28	18.19 ±3.30	394.06 ±20.00	20.49 ±2.67	52.23 ±4.54	9.40 ±0.54	*22580.52*
Limit of detection	*1.47*	*0.04*	*0.32*	*0.07*	*0.10*	*0.19*	*0.06*	*2.30*	*0.83*	*1.65*	*0.60*	*0.05*	*0.01*	
Limit of quantification	*4.45*	*0.11*	*0.99*	*0.21*	*0.31*	*0.56*	*0.17*	*6.96*	*2.51*	*5.01*	*1.83*	*0.14*	*0.03*	

*Table S5. PFAS concentrations (pg/m^3) and Na^+ levels (µg/ m^3) at the Hazelrigg observatory during the sampling campaign. Levels between MDL and MQL are in italic and marked by and asterisk**

Sample	PFBA	PFPeA	PFHxA	PFHpA	PFOA	PFNA	PFDA	PFUnDA	PFDoDA	PFTriDA	PFTeDA	PFBS	PFHxS	PFOS	6:2 FTS	EEA	Na^+	ΣPFAS
HAZ-1	<MDL	<MDL	<MDL	<MDL	0.86	0.04*	0.03	0.02	0.02*	<MDL	<MDL	<MDL	0.007*	0.16	0.12	<MDL	9.77	1.23
HAZ-2	<MDL	<MDL	<MDL	<MDL	0.90	<MDL	0.02*	0.01*	<MDL	<MDL	<MDL	<MDL	0.002*	0.18	0.04*	<MDL	4.60	1.16
HAZ-3	<MDL	<MDL	<MDL	<MDL	<MDL	<MDL	<MDL	<MDL	<MDL	<MDL	<MDL	<MDL	0.003*	0.22	0.06*	<MDL	0.65	0.29
HAZ-4	<MDL	<MDL	<MDL	<MDL	0.52*	<MDL	0.02*	<MDL	<MDL	<MDL	<MDL	<MDL	0.003*	0.19	0.05*	<MDL	1.68	0.78
HAZ-5	<MDL	<MDL	<MDL	<MDL	0.77	<MDL	0.03*	0.02*	<MDL	<MDL	<MDL	<MDL	0.003*	0.49	0.05*	<MDL	3.94	1.37
HAZ-6	<MDL	<MDL	<MDL	<MDL	1.02	<MDL	0.04*	<MDL	0.01*	<MDL	<MDL	<MDL	0.005*	0.36	0.06*	<MDL	3.54	1.50
HAZ-7	<MDL	<MDL	<MDL	<MDL	0.67*	<MDL	<MDL	<MDL	<MDL	<MDL	<MDL	<MDL	0.005*	0.13	0.04*	<MDL	2.37	0.85
HAZ-8	<MDL	<MDL	<MDL	<MDL	0.75	<MDL	0.04*	<MDL	0.01*	<MDL	<MDL	<MDL	0.003*	0.14	0.08	<MDL	1.69	1.04
HAZ-9	<MDL	<MDL	<MDL	<MDL	1.14	<MDL	0.04	0.02	0.01*	<MDL	<MDL	<MDL	0.003*	0.17	0.04*	<MDL	3.67	1.44
HAZ-10	<MDL	<MDL	<MDL	<MDL	0.69	<MDL	0.02	<MDL	<MDL	<MDL	<MDL	<MDL	0.002*	0.12	0.07	<MDL	1.79	0.91
HAZ-11	<MDL	<MDL	<MDL	<MDL	1.52	0.12*	0.08	0.07	0.03	0.02*	<MDL	<MDL	0.007*	0.20	0.04*	<MDL	4.59	2.10
HAZ-12	<MDL	<MDL	<MDL	<MDL	1.36	<MDL	0.02*	0.01*	<MDL	<MDL	<MDL	<MDL	0.004*	0.13	0.03*	<MDL	3.63	1.57
HAZ-13	<MDL	<MDL	<MDL	<MDL	2.41	<MDL	0.02*	0.01*	<MDL	<MDL	<MDL	<MDL	0.006*	0.18	0.08*	<MDL	2.35	2.72
HAZ-14	0.22*	<MDL	0.10*	0.12	2.50	0.10	0.04	0.03	0.02	<MDL	<MDL	<MDL	0.014*	0.20	0.06	2.03	14.64	5.43
HAZ-15	0.32*	<MDL	0.08*	0.10	2.50	0.08	0.04	0.02	0.01	<MDL	<MDL	<MDL	0.011*	0.14	0.03*	0.28*	13.03	3.61
HAZ-16	<MDL	<MDL	<MDL	<MDL	1.63	<MDL	0.02*	<MDL	<MDL	<MDL	<MDL	<MDL	0.002*	0.29	0.07*	<MDL	0.97	2.02
HAZ-17	<MDL	<MDL	<MDL	<MDL	1.70	<MDL	0.02	0.01*	0.01*	<MDL	<MDL	<MDL	0.001*	0.16	0.04*	<MDL	3.30	1.94
HAZ-18	<MDL	<MDL	<MDL	<MDL	1.53	<MDL	0.03	0.03	<MDL	<MDL	<MDL	<MDL	0.003*	0.12	0.04*	<MDL	4.40	1.75
HAZ-19	<MDL	<MDL	<MDL	<MDL	1.00	<MDL	0.04	0.01*	0.01*	<MDL	<MDL	<MDL	0.006*	0.09	0.09*	<MDL	1.41	1.25
HAZ-20	<MDL	<MDL	<MDL	<MDL	1.66	<MDL	0.02*	0.01*	<MDL	<MDL	<MDL	<MDL	0.003*	0.10	0.05	<MDL	3.27	1.83
HAZ-21	<MDL	<MDL	0.03*	0.08*	2.21	0.08	0.03	0.02	0.01*	<MDL	<MDL	<MDL	0.008*	0.12	0.06	0.67	8.91	3.31

Table S6. Method limits of detection (MDL, pg/m^3) for all air samples

MDL	PFBA	PFPeA	PFHxA	PFHpA	PFOA	PFNA	PFDA	PFUnDA	PFDoDA	PFTriDA	PFTeDA	PFBS	PFHxS	PFOS	6:2 FTS	EEA
HAZ-1	0.65	0.15	0.03	0.03	0.02	0.03	0.01	0.01	0.01	0.02	0.03	0.07	0.002	0.003	0.02	0.19
HAZ-2	1.62	0.34	0.08	0.11	0.06	0.07	0.01	0.01	0.01	0.02	0.03	0.06	0.002	0.002	0.02	0.58
HAZ-3	No IS	78.99	No IS	10.90	1.03	1.58	0.07	0.04	0.03	0.08	0.12	0.07	0.002	0.003	0.02	59.63
HAZ-4	39.70	1.37	0.24	0.44	0.20	0.23	0.02	0.01	0.01	0.03	0.04	0.06	0.002	0.002	0.02	2.40
HAZ-5	2.57	0.47	0.11	0.15	0.09	0.12	0.01	0.01	0.01	0.04	0.05	0.08	0.002	0.003	0.03	0.83
HAZ-6	25.22	1.22	0.27	0.33	0.17	0.19	0.01	0.01	0.01	0.03	0.04	0.07	0.002	0.002	0.03	1.81
HAZ-7	23.77	9.65	0.54	0.84	0.35	0.42	0.03	0.02	0.02	0.05	0.07	0.08	0.002	0.003	0.03	4.59
HAZ-8	5.15	0.48	0.12	0.18	0.11	0.15	0.01	0.01	0.01	0.04	0.05	0.07	0.002	0.003	0.03	1.01
HAZ-9	0.94	0.25	0.06	0.07	0.04	0.05	0.01	0.01	0.01	0.01	0.02	0.08	0.002	0.002	0.02	0.41
HAZ-10	0.87	0.18	0.04	0.06	0.04	0.06	0.01	0.01	0.01	0.02	0.03	0.06	0.002	0.002	0.02	0.33
HAZ-11	1.54	0.43	0.09	0.15	0.08	0.08	0.01	0.01	0.01	0.01	0.02	0.06	0.002	0.002	0.02	0.82
HAZ-12	2.03	0.35	0.07	0.11	0.06	0.08	0.01	0.01	0.01	0.02	0.02	0.05	0.002	0.002	0.02	0.60
HAZ-13	2.87	0.75	0.20	0.29	0.15	0.15	0.01	0.01	0.01	0.03	0.04	0.11	0.002	0.004	0.04	1.61
HAZ-14	0.20	0.05	0.02	0.02	0.01	0.02	0.01	0.01	0.01	0.01	0.01	0.06	0.002	0.002	0.02	0.10
HAZ-15	0.30	0.07	0.02	0.02	0.01	0.02	0.01	0.01	0.01	0.01	0.01	0.05	0.002	0.002	0.02	0.12
HAZ-16	10.44	0.52	0.12	0.18	0.12	0.15	0.01	0.01	0.01	0.03	0.04	0.07	0.002	0.002	0.03	0.97
HAZ-17	0.51	0.16	0.04	0.06	0.04	0.04	0.01	0.01	0.01	0.01	0.02	0.06	0.002	0.002	0.02	0.31
HAZ-18	0.81	0.22	0.05	0.08	0.05	0.08	0.01	0.01	0.01	0.02	0.02	0.06	0.002	0.002	0.02	0.43
HAZ-19	0.87	0.21	0.06	0.09	0.07	0.10	0.01	0.01	0.01	0.02	0.03	0.06	0.002	0.002	0.02	0.50
HAZ-20	7.12	0.88	0.19	0.20	0.10	0.10	0.01	0.01	0.01	0.02	0.02	0.05	0.002	0.002	0.02	1.11
HAZ-21	0.30	0.08	0.02	0.02	0.02	0.03	0.01	0.01	0.01	0.01	0.01	0.06	0.002	0.002	0.02	0.14

Table S7. Method limits of quantification (MQL, pg/m³) for all air samples

MQL	PFBA	PFPeA	PFHxA	PFHpA	PFOA	PFNA	PFDA	PFUnDA	PFDoDA	PFTriDA	PFTeDA	PFBS	PFHxS	PFOS	6:2 FTS	EEA
HAZ-1	1.50	0.46	0.29	0.12	0.09	0.08	0.01	0.02	0.02	0.08	0.09	0.07	0.03	0.07	0.06	0.80
HAZ-2	3.73	1.09	0.93	0.37	0.25	0.18	0.02	0.02	0.02	0.07	0.09	0.07	0.03	0.006	0.06	2.47
HAZ-3	No IS	251.75	No IS	38.17	4.37	3.89	0.26	0.12	0.07	0.30	0.36	0.08	0.03	0.008	0.07	255.50
HAZ-4	91.07	4.35	2.95	1.53	0.85	0.57	0.06	0.04	0.02	0.10	0.12	0.06	0.03	0.006	0.05	10.24
HAZ-5	5.91	1.50	1.36	0.53	0.39	0.30	0.04	0.04	0.03	0.13	0.16	0.09	0.03	0.008	0.09	3.54
HAZ-6	57.86	3.87	3.35	1.15	0.70	0.47	0.05	0.04	0.02	0.09	0.11	0.08	0.03	0.006	0.08	7.72
HAZ-7	54.52	30.74	6.76	2.93	1.47	1.03	0.11	0.07	0.04	0.18	0.22	0.09	0.03	0.008	0.08	19.64
HAZ-8	11.81	1.54	1.47	0.64	0.46	0.38	0.05	0.04	0.03	0.13	0.15	0.08	0.03	0.007	0.07	4.31
HAZ-9	2.15	0.80	0.68	0.26	0.19	0.13	0.02	0.02	0.01	0.05	0.06	0.09	0.03	0.007	0.06	1.73
HAZ-10	1.99	0.56	0.40	0.21	0.18	0.15	0.02	0.02	0.01	0.07	0.08	0.06	0.02	0.005	0.06	1.41
HAZ-11	3.55	1.36	1.08	0.52	0.33	0.21	0.02	0.02	0.01	0.05	0.06	0.07	0.03	0.006	0.05	3.46
HAZ-12	4.65	1.11	0.85	0.38	0.27	0.19	0.02	0.02	0.01	0.06	0.08	0.05	0.02	0.005	0.05	2.54
HAZ-13	6.59	2.38	2.36	1.02	0.62	0.38	0.04	0.03	0.02	0.10	0.11	0.12	0.05	0.010	0.10	6.84
HAZ-14	0.46	0.17	0.14	0.06	0.06	0.05	0.01	0.01	0.01	0.03	0.04	0.06	0.03	0.005	0.05	0.42
HAZ-15	0.69	0.24	0.17	0.07	0.06	0.05	0.01	0.01	0.01	0.03	0.03	0.05	0.03	0.005	0.04	0.47
HAZ-16	23.96	1.64	1.39	0.61	0.50	0.37	0.04	0.03	0.02	0.10	0.11	0.07	0.03	0.006	0.07	4.11
HAZ-17	1.18	0.52	0.44	0.19	0.15	0.11	0.02	0.01	0.01	0.04	0.05	0.07	0.03	0.006	0.06	1.31
HAZ-18	1.86	0.71	0.55	0.27	0.23	0.19	0.03	0.02	0.01	0.06	0.07	0.06	0.03	0.006	0.05	1.82
HAZ-19	2.00	0.68	0.65	0.32	0.30	0.25	0.03	0.03	0.02	0.08	0.09	0.06	0.03	0.006	0.06	2.13
HAZ-20	16.34	2.81	2.37	0.71	0.43	0.25	0.03	0.02	0.01	0.06	0.07	0.06	0.03	0.005	0.06	4.73
HAZ-21	0.70	0.24	0.21	0.09	0.08	0.07	0.01	0.01	0.01	0.04	0.04	0.07	0.03	0.006	0.06	0.58

Table S8. IS recoveries (%) for the air samples; shown are the detected targets with an internal standard

IS-Rec	PFBA	PFPeA	PFHxA	PFHpA	PFOA	PFNA	PFDA	PFUnDA	PFDoDA	PFHxS	PFOS	6:2 FTS
HAZ-1	11.34%	12.53%	11.66%	17.83%	24.57%	28.54%	28.23%	24.60%	15.66%	38.12%	36.12%	65.07%
HAZ-2	5.73%	6.60%	4.53%	7.12%	11.42%	16.45%	21.39%	25.87%	21.22%	43.56%	45.83%	67.47%
HAZ-3	No IS	0.03%	No IS	0.07%	0.62%	0.72%	1.88%	4.08%	4.94%	41.45%	40.89%	66.62%
HAZ-4	0.20%	1.43%	1.22%	1.48%	2.87%	4.45%	7.96%	11.87%	13.59%	47.24%	47.59%	83.57%
HAZ-5	5.00%	6.66%	4.27%	6.87%	10.00%	13.45%	17.29%	19.21%	16.26%	44.04%	43.96%	61.90%
HAZ-6	0.46%	2.30%	1.54%	2.81%	4.98%	7.76%	13.22%	17.44%	20.52%	43.38%	49.97%	63.89%
HAZ-7	0.39%	0.23%	0.62%	0.89%	1.91%	2.85%	4.73%	7.68%	8.49%	38.12%	39.78%	58.01%
HAZ-8	2.06%	5.33%	3.25%	4.64%	7.06%	8.79%	11.75%	15.59%	13.72%	40.50%	42.53%	62.74%
HAZ-9	11.35%	10.28%	7.14%	11.69%	17.54%	24.99%	30.39%	36.23%	34.45%	39.72%	46.86%	72.04%
HAZ-10	9.80%	11.77%	9.59%	11.42%	14.56%	17.79%	20.12%	22.95%	21.55%	40.23%	43.56%	56.00%
HAZ-11	4.45%	3.92%	2.88%	3.75%	6.40%	10.30%	16.60%	22.33%	22.09%	37.24%	41.63%	65.12%
HAZ-12	3.09%	4.35%	3.32%	4.65%	7.06%	10.19%	14.83%	17.78%	16.40%	41.62%	42.56%	63.56%
HAZ-13	4.44%	4.15%	2.44%	3.52%	6.26%	10.59%	16.23%	21.46%	22.32%	39.31%	44.39%	66.37%
HAZ-14	37.08%	34.62%	25.60%	34.34%	41.19%	47.44%	49.66%	49.81%	41.65%	38.63%	45.05%	66.69%
HAZ-15	23.09%	22.99%	19.34%	28.62%	36.72%	44.06%	48.04%	50.74%	47.07%	44.82%	50.53%	79.53%
HAZ-16	1.04%	5.12%	3.52%	4.98%	6.65%	9.19%	13.53%	18.28%	18.79%	46.36%	49.28%	67.33%
HAZ-17	17.02%	13.06%	9.03%	12.79%	18.36%	25.16%	31.29%	37.76%	37.31%	44.54%	48.12%	73.42%
HAZ-18	9.29%	8.24%	6.28%	7.85%	10.21%	12.61%	16.44%	21.49%	21.85%	45.26%	49.00%	74.49%
HAZ-19	9.37%	9.27%	5.69%	7.26%	8.20%	10.26%	12.80%	16.86%	17.55%	41.76%	44.11%	56.92%
HAZ-20	1.31%	2.56%	1.76%	3.72%	6.56%	11.80%	17.84%	25.43%	28.44%	47.69%	52.26%	65.02%
HAZ-21	29.08%	28.87%	19.40%	29.43%	34.87%	42.29%	44.84%	45.30%	41.23%	40.31%	44.70%	61.90%

Table S9. Mean recoveries of internal standards from both air and water sample extraction methods. Due to the suppression of PFOA responses due to high concentrations, M8PFOS was used as the recovery standard for most WAX-SPE internal standards

Internal standard	Mean Recovery-QFFs (air samples)	Based on recovery standard	WAX-SPE	Based on recovery standard
**M4PFBA**	27.32%	M8PFOA	20.88%	M8PFOS
**M5PFPeA**	23.22%	M8PFOA	92.95%	M8PFOS
**M2PFHxA**	17.97%	M8PFOA	49.44%	M8PFOS
**M4PFHpA**	19.17%	M8PFOA	86.94%	M8PFOS
**M4PFOA**	20.72%	M8PFOA	60.62%	M8PFOA
**M5PFNA**	25.56%	M8PFOA	73.66%	M8PFOS
**M2PFDA**	26.34%	M8PFOA	43.51%	M8PFOS
**M2PFUnDA**	29.03%	M8PFOA	41.08%	M8PFOS
**M2PFDoDA**	26.77%	M8PFOA	25.50%	M8PFOS
**MPFHxS**	43.91%	M8PFOS	68.23%	M8PFOS
**MPFOS**	46.39%	M8PFOS	48.41%	M8PFOS
**M2-6:2FTS**	75.89%	M8PFOS	170.80%	M8PFOS
**M3-HFPO-DA**	14.50%	M8PFOA	36.91%	M8PFOS
**d3-MeFOSAA**	42.51%	M8PFOS	110.29%	M8PFOS
**d5-EtFOSAA**	36.17%	M8PFOS	84.81%	M8PFOS
**M8-FOSA**	Not found	-	1.39%	M8PFOS

Table S10. Characteristics of the identified suspects. Substances in italic have an isomeric calibration standard.

Class	Name	m/z	Mass error (ppm)	RT	MS^2	Confidence level	Formula	In-source fragment detected
**1. H-PFCAs**	H-PFBA	194.9880	1.86	0.60	No	3	$C_4H_2F_6O_2$	-
	H-PFPeA	244.9848	1.11	0.94	For $[M-CO_2F-H]^-$	2b	$C_5H_2F_8O_2$	$[M-CO_2F-H]$-
	H-PFHxA	294.9816	1.69	1.81	Yes	2b	$C_6H_2F_{10}O_2$	$[M-CO_2F-H]^-$
	H-PFHpA	344.9784	1.66	2.80	Yes	2b	$C_7H_2F_{12}O_2$	$[M-CO_2F-H]^-$
	H-PFOA	394.9753	1.34	3.35	Yes	1	$C_8H_2F_{14}O_2$	$[M-CO_2F-H]^-$
	H-PFNA	444.9721	1.11	3.76	Yes	1	$C_9H_2F_{16}O_2$	$[M-CO_2F-H]^-$
	H-PFDA	494.9689	0.97	4.12	Yes	2b	$C_{10}H_2F_{18}O_2$	$[M-CO_2F-H]^-$
	H-PFUnDA	544.9657	0.74	4.45	Yes	1	$C_{11}H_2F_{20}O_2$	-
	H-PFDoDA	594.9625	2.37	4.88	No	3	$C_{12}H_2F_{22}O_2$	-
**2. Cl-PFCAs**	Cl-PFBA	228.9491	1.18	0.87	No	3	$C_4HClF_6O_2$	-
	Cl-PFPeA	278.9459	1.14	2.00	No	3	$C_5HClF_8O_2$	-
	Cl-PFHxA	328.9427	1.69	2.95	No	3	$C_6HClF_{10}O_2$	$[M-CO_2-H]^-$
	Cl-PFHpA	378.9395	1.67	3.52	No	3	$C_7HClF_{12}O_2$	$[M-CO_2-H]^-$
	Cl-PFOA	428.9363	1.23	3.96	No	3	$C_8HClF_{14}O_2$	$[M-CO_2-H]^-$
	Cl-PFNA	478.9331	1.16	4.37	No	3	$C_9HClF_{16}O_2$	$[M-CO_2-H]^-$
	Cl-PFDA	528.9299	0.33	4.73	No	3	$C_{10}HClF_{18}O_2$	$[M-CO_2-H]^-$
	Cl-PFUnDA	578.9267	2.56	5.10	No	3	$C_{11}HClF_{20}O_2$	$[M-CO_2-H]^-$
	Cl-PFDoDA	628.9235		5.49	No	3	$C_{12}HClF_{22}O_2$	$[M-CO_2-H]^-$
**3. ME-PFECAs**	*Ether-PFBA*	228.9735	1.25	1.03	Yes	2b	$C_4HF_7O_3$	-
	Ether-PFPeA	278.9703	0.43	2.10	No	3	$C_5HF_9O_3$	$C_nF_{2n+1}O^-$
	Ether-PFHxA	328.9671	1.89	3.01	No	3	$C_6HF_{11}O_3$	$C_nF_{2n+1}O^-$
	Ether-PFHpA	378.9639	1.96	3.60	Yes	2b	$C_7HF_{13}O_3$	$C_nF_{2n+1}O^-$
	Ether-PFOA	428.9607	1.27	4.05	Yes	2b	$C_8HF_{15}O_3$	$C_nF_{2n+1}O^-$
	Ether-PFNA	478.9576	1.36	4.31	No	3	$C_9HF_{17}O_3$	$C_nF_{2n+1}O^-$
**4. Cyc. ME-PFECAs**	Cyc. Ether-PFPeA	240.9735	1.38	1.10	For $[M-CO_2-H]^-$	2b	$C_5HF_7O_3$	$[M-CO_2-H]^-$
	Cyc. Ether PFHxA	290.9703	1.35	2.37	For [M-CO2-H]-	2b	$C_6HF_9O_3$	$[M-CO_2-H]^-$

	Cyc. Ether PFHpA	340.9671	2.19	3.01	Yes	2b	$C_7HF_{11}O_3$	$[M-CO_2-H]^-$
	Cyc. Ether PFOA	390.9639	1.27	3.57	Yes	2b	$C_8HF_{13}O_3$	$[M-CO_2-H]^-$
**5. 6:2 FTA + structural isomers**	6:2 FTA/H2-PFOA	376.9847	2.16	2.94-3.57 (multiple peaks)	Yes	2a	$C_8H_3F_{13}O_2$	$[M-CO_2F_2-H_3]^-$
**6. H-substituted structures of class 2. and 3.**	H-Ether-PFPeA	260.9797	1.31	2.14	No	3	$C_5H_2F_8O_3$	-
	H-Ether-PFHxA	310.9766	2.23	2.66	No	3	$C_6H_2F_{10}O_3$	-
	H-Ether-PFHpA	360.9734	2.14	3.14	No	3	$C_7H_2F_{12}O_3$	-
	H-Ether-PFOA	410.9702	2.16	3.65	No	3	$C_8H_2F_{14}O_3$	-
	H-Ether-PFNA	460.9670	1.63	4.04	No	3	$C_9H_2F_{16}O_3$	-
	H-Ether-PFDA	510.9638	1.35	4.36	No	3	$C_{10}H_2F_{18}O_3$	-
	H-Cl-PFHxA	310.9521	0.84	2.65	No	3	$C_6H_2ClF_9O_2$	-
	H-Cl-PFHpA	360.9489	1.70	3.42	No	3	$C_7H_2ClF_{11}O_2$	-
	H-Cl-PFOA	410.9457	2.35	3.80	No	3	$C_8H_2ClF_{13}O_2$	-
	H-Cl-PFNA	460.9425	1.42	4.10	No	3	$C_9H_2ClF_{15}O_2$	-
	H-Cl-PFDA	510.9393	1.08	4.46	No	3	$C_{10}H_2ClF_{17}O_2$	-
**7. Multiple H-substituted PFCAs**	H3-PFHxA	259.0005	3.60	2.19	No	3	$C_6H_4F_8O_2$	-
	H3-PFOA	358.9941	2.21	3.11	Yes	2b	$C_8H_4F_{12}O_2$	$[M-CO_2F_3-H_4]$-
	H4-PFOA	341.0035	2.36	3.13	Yes	2b	$C_8H_5F_{11}O_2$	$[M-CO_2F_4-H_5]$-
	H4-PFNA	391.0003	1.08	3.72	Yes	2b	$C_9H_5F_{13}O_2$	$[M-CO_2F_4-H_5]$-
	H4-PFDA	440.9971	1.20	4.06	Yes	2b	$C_{10}H_5F_{15}O_2$	$[M-CO_2F_4-H_5]$-
	H4-PFUnDA	490.9939	0.76	4.42	No	3	$C_{11}H_5F_{17}O_2$	-
	H4-PFDoDA	540.9907	0.55	4.76	No	3	$C_{12}H_5F_{19}O_2$	-
	H5-PFHpA	273.0161	1.09	1.23	No	3	$C_7H_6F_8O_2$	-
	H5-PFOA	323.0129	1.76	2.39	No	3	$C_8H_6F_{10}O_2$	-
	H5-PFNA	373.0097	2.14	3.20	Yes	2b	$C_9H_6F_{12}O_2$	$[M-CO_2F_5H_6]^-$
	H5-PFDA	423.0066	1.35	3.59	No	3	$C_{10}H_6F_{14}O_2$	-
	H5-PFUnDA	473.0034	1.39	3.95	No	3	$C_{11}H_6F_{16}O_2$	-
	H5-PFDoDA	523.0002	1.23	4.52	No	3	$C_{12}H_6F_{18}O_2$	-
	H5-PFTriDA	572.9970	3.70	5.00	No	3	$C_{13}H_6F_{20}O_2$	-
	H5-PFTeDA	622.9938	2.08	5.43	No	3	$C_{14}H_6F_{22}O_2$	-
	H5-PFPeDA	672.9906	3.67	5.61	No	3	$C_{15}H_6F_{24}O_2$	-

Table S11. Response areas of the suspects in the water samples. A response area above 1e8 is marked red, between 1e7 and 1e8 dark yellow, between 1e6 and 1e7 light yellow and between 1e4 and 1e6 light green. N/F means "not found'. A different injection series than the quantification injections was used to acquire the response areas shown here.

Class	Name	HLP	WMD-1	WMD-2	WLD	WLPD	DBL-1	DBL-2
1. **H-PFCAs**	H-PFBA	3.9E+07	N/F	N/F	1.7E+06	2.8E+07	1.8E+08	1.7E+08
	H-PFPeA	2.9E+07	N/F	5.2E+06	4.1E+06	5.1E+07	2.3E+08	1.9E+08
	H-PFHxA	2.8E+07	9.8E+04	1.6E+05	3.0E+06	3.3E+07	4.0E+08	3.5E+08
	H-PFHpA	1.8E+07	6.7E+05	N/F	5.9E+06	4.6E+07	3.2E+08	3.0E+08
	H-PFOA	1.9E+07	2.9E+06	2.1E+06	6.4E+06	4.5E+08	8.8E+08	7.9E+08
	H-PFNA	1.8E+07	3.9E+06	3.2E+06	3.5E+06	6.5E+07	2.5E+08	2.2E+08
	H-PFDA	2.7E+07	4.7E+06	4.4E+06	2.1E+06	7.7E+07	2.2E+08	1.7E+08
	H-PFUnDA	1.2E+07	4.9E+05	4.8E+05	6.1E+05	4.7E+06	3.0E+07	2.7E+07
	H-PFDoDA	6.6E+06	1.2E+05	1.4E+05	4.8E+05	1.7E+06	2.3E+06	2.5E+06
2. **Cl-PFCAs**	Cl-PFBA	1.4E+07	N/F	N/F	7.0E+06	1.7E+07	9.1E+07	8.0E+07
	Cl-PFPeA	1.3E+07	1.1E+04	N/F	1.0E+07	4.0E+07	1.6E+08	1.4E+08
	Cl-PFHxA	2.0E+07	3.0E+05	6.9E+04	2.1E+07	1.3E+08	4.0E+08	3.6E+08
	Cl-PFHpA	1.7E+07	8.5E+05	5.5E+05	1.2E+07	1.5E+08	4.4E+08	3.9E+08
	Cl-PFOA	4.0E+07	4.9E+06	4.0E+06	2.5E+07	3.9E+08	7.3E+08	6.7E+08
	Cl-PFNA	2.6E+07	1.9E+06	1.8E+06	1.3E+07	9.6E+07	1.1E+08	9.1E+07
	Cl-PFDA	3.8E+07	5.7E+05	6.6E+05	7.5E+06	1.8E+07	2.6E+07	2.2E+07
	Cl-PFUnDA	9.3E+06	7.4E+04	N/F	9.2E+05	2.5E+06	7.5E+05	1.1E+06

	Cl-PFDoDA	1.5E+06	N/F	N/F	1.1E+05	1.5E+05	N/F	6.7E+04
**3. ME-PFECAs**	*Ether-PFBA*	3.7E+07	8.8E+05	4.3E+05	2.7E+07	1.7E+07	9.2E+06	8.3E+06
	Ether-PFPeA	1.9E+06	N/F	N/F	1.6E+06	3.6E+06	3.3E+06	1.9E+06
	Ether-PFHxA	4.8E+05	N/F	N/F	6.6E+05	5.3E+06	8.2E+06	6.1E+06
	Ether-PFHpA	1.1E+06	N/F	N/F	9.0E+05	1.1E+07	1.7E+07	1.3E+07
	Ether-PFOA	2.2E+07	5.4E+04	N/F	2.1E+07	1.2E+08	1.8E+08	1.3E+08
	Ether-PFNA	7.0E+06	N/F	N/F	6.2E+06	2.0E+07	2.4E+07	1.9E+07
**4. Cyc. ME-PFECAs**	Cyc. Ether-PFBA	N/F	N/F	N/F	N/F	N/F	N/F	N/F
	Cyc. Ether-PFPeA	9.4E+04	N/F	N/F	N/F	2.8E+07	4.1E+07	3.6E+07
	Cyc. Ether PFHxA	2.7E+05	N/F	N/F	N/F	2.2E+07	3.0E+07	2.5E+07
	Cyc. Ether PFHpA	8.1E+05	N/F	N/F	2.3E+05	2.3E+07	3.8E+07	3.1E+07
	Cyc. Ether PFOA	4.3E+07	N/F	N/F	2.0E+07	4.7E+08	7.3E+08	6.0E+08
**5. 6:2 FTA + structural isomers**	6:2 FTA/H2-PFOA	2.5E+06	2.6E+05	1.2E+05	5.1E+05	3.9E+08	1.0E+08	9.4E+07
**6. H-substituted structures of class 2. and 3.**	H-Ether-PFPeA	N/F	N/F	N/F	N/F	8.8E+04	8.3E+05	6.6E+05
	H-Ether-PFHxA	N/F	N/F	N/F	N/F	8.5E+05	1.5E+06	1.2E+06
	H-Ether-PFHpA	N/F	N/F	N/F	N/F	1.5E+06	2.5E+06	2.1E+06
	H-Ether-PFOA	1.2E+05	1.2E+05	N/F	N/F	2.8E+06	6.1E+06	4.6E+06
	H-Ether-PFNA	N/F	N/F	N/F	N/F	N/F	2.0E+05	1.1E+05
	H-Ether-PFDA	1.3E+04	N/F	N/F	N/F	1.9E+05	2.8E+05	1.9E+05

	H-Cl-PFHxA	N/F	N/F	N/F	N/F	7.3E+04	1.9E+06	1.8E+06
	H-Cl-PFHpA	N/F	N/F	N/F	N/F	1.3E+05	8.1E+05	6.2E+05
	H-Cl-PFOA	1.4E+05	N/F	N/F	N/F	1.2E+06	3.1E+06	2.7E+06
	H-Cl-PFNA	N/F	N/F	N/F	N/F	2.6E+05	8.7E+05	4.6E+05
	H-Cl-PFDA	2.1E+05	N/F	N/F	N/F	1.8E+05	3.1E+05	2.8E+05
**8. Multiple H-substituted PFCAs**	H3-PFHxA	N/F	N/F	N/F	N/F	N/F	N/F	N/F
	H3-PFOA	N/F	N/F	N/F	N/F	3.1E+07	9.5E+06	7.7E+06
	H4-PFOA	1.0E+07	3.1E+05	4.4E+05	5.0E+05	1.4E+07	5.1E+06	4.3E+06
	H4-PFNA	3.7E+07	5.0E+05	5.0E+05	3.0E+06	4.3E+05	3.9E+05	N/F
	H4-PFDA	5.3E+06	1.4E+05	N/F	2.8E+05	1.3E+05	2.8E+05	1.9E+05
	H4-PFUnDA	2.7E+06	N/F	N/F	N/F	N/F	N/F	N/F
	H4-PFDoDA	5.3E+05	N/F	N/F	N/F	N/F	N/F	N/F
	H5-PFHpA	3.7E+06	N/F	N/F	N/F	N/F	1.5E+05	1.5E+05
	H5-PFOA	6.3E+05	N/F	N/F	N/F	6.4E+04	N/F	N/F
	H5-PFNA	5.9E+06	N/F	N/F	3.8E+04	1.9E+04	N/F	5.3E+05
	H5-PFDA	3.1E+05	N/F	N/F	N/F	N/F	N/F	N/F
	H5-PFUnDA	3.1E+05	N/F	N/F	N/F	N/F	N/F	N/F
	H5-PFDoDA	1.3E+05	N/F	N/F	N/F	N/F	N/F	N/F
	H5-PFTriDA	1.5E+04	N/F	N/F	N/F	N/F	N/F	N/F

	H5-PFTeDA	8.4E+03	N/F	N/F	N/F	N/F	N/F	N/F
	H5-PFPeDA	7.5E+04	5.7E+04	2.5E+04	6.4E+04	9.5E+04	N/F	N/F

Table S12. Response areas of suspects normalized by the average response area of the C_4-C_{12} PFCA IS. Normalized areas above 0.001 are marked light green, between 0.01 and 0.1 yellow, between 0.1 and 1.0 orange and above 1.0 red. N/F means "not found".

Class	Name	HLP	WMD-1	WMD-2	WLD	WLPD	DBL-1	DBL-2
**1. H-PFCAs**	H-PFBA	0.978	N/F	N/F	0.033	0.691	5.223	5.694
	H-PFPeA	0.712	N/F	0.099	0.080	1.275	6.479	6.324
	H-PFHxA	0.703	0.002	0.003	0.058	0.829	11.389	11.371
	H-PFHpA	0.452	0.010	N/F	0.115	1.157	9.051	9.837
	H-PFOA	0.468	0.045	0.040	0.126	11.218	25.119	26.016
	H-PFNA	0.437	0.061	0.062	0.069	1.612	7.082	7.281
	H-PFDA	0.661	0.073	0.084	0.040	1.929	6.211	5.716
	H-PFUnDA	0.305	0.008	0.009	0.012	0.118	0.860	0.897
	H-PFDoDA	0.164	0.002	0.003	0.009	0.041	0.066	0.082
**2. Cl-PFCAs**	Cl-PFBA	0.339	N/F	N/F	0.137	0.433	2.597	2.607
	Cl-PFPeA	0.316	0.000	N/F	0.195	0.998	4.628	4.701
	Cl-PFHxA	0.490	0.005	0.001	0.408	3.329	11.401	11.819
	Cl-PFHpA	0.418	0.013	0.011	0.239	3.823	12.641	12.600

	Cl-PFOA	0.997	0.077	0.077	0.493	9.634	20.787	21.774
	Cl-PFNA	0.655	0.030	0.035	0.255	2.403	3.267	2.987
	Cl-PFDA	0.960	0.009	0.013	0.148	0.440	0.727	0.734
	Cl-PFUnDA	0.233	0.001	N/F	0.018	0.063	0.021	0.037
	Cl-PFDoDA	0.038	N/F	N/F	0.002	0.004	N/F	0.002
**3. ME-PFECAs**	*Ether-PFBA*	0.918	0.014	0.008	0.533	0.419	0.261	0.270
	Ether-PFPeA	0.048	N/F	N/F	0.032	0.090	0.092	0.061
	Ether-PFHxA	0.012	N/F	N/F	0.013	0.133	0.234	0.200
	Ether-PFHpA	0.027	N/F	N/F	0.018	0.270	0.480	0.419
	Ether-PFOA	0.558	0.001	N/F	0.415	3.062	4.981	4.332
	Ether-PFNA	0.175	N/F	N/F	0.122	0.490	0.678	0.617
**4. Cyc. ME-PFECAs**	Cyc. Ether-PFBA	N/F	N/F	N/F	N/F	N/F	N/F	N/F
	Cyc. Ether-PFPeA	0.002	N/F	N/F	N/F	0.697	1.156	1.172
	Cyc. Ether PFHxA	0.007	N/F	N/F	N/F	0.549	0.853	0.817
	Cyc. Ether PFHpA	0.020	N/F	N/F	0.005	0.583	1.071	1.013
	Cyc. Ether PFOA	1.065	N/F	N/F	0.384	11.829	20.875	19.483
**5. 6:2 FTA + structural isomers**	6:2 FTA/H2-PFOA	0.063	0.004	0.002	0.010	9.696	2.934	3.070
**6. H-substituted structures of class 2. and 3.**	H-Ether-PFPeA	N/F	N/F	N/F	N/F	0.002	0.024	0.021
	H-Ether-PFHxA	N/F	N/F	N/F	N/F	0.021	0.044	0.039

	H-Ether-PFHpA	N/F	N/F	N/F	N/F	0.037	0.072	0.069
	H-Ether-PFOA	0.003	0.002	N/F	N/F	0.071	0.173	0.152
	H-Ether-PFNA	N/F	N/F	N/F	N/F	N/F	0.006	0.004
	H-Ether-PFDA	0.000	N/F	N/F	N/F	0.005	0.008	0.006
	H-Cl-PFHxA	N/F	N/F	N/F	N/F	0.002	0.054	0.060
	H-Cl-PFHpA	N/F	N/F	N/F	N/F	0.003	0.023	0.020
	H-Cl-PFOA	0.004	N/F	N/F	N/F	0.029	0.089	0.088
	H-Cl-PFNA	N/F	N/F	N/F	N/F	0.007	0.025	0.015
	H-Cl-PFDA	0.005	N/F	N/F	N/F	0.005	0.009	0.009
**9. Multiple H-substituted PFCAs**	H3-PFHxA	N/F	N/F	N/F	N/F	N/F	N/F	N/F
	H3-PFOA	N/F	N/F	N/F	N/F	0.774	0.271	0.253
	H4-PFOA	0.255	0.005	0.008	0.010	0.353	0.145	0.140
	H4-PFNA	0.930	0.008	0.009	0.059	0.011	0.011	N/F
	H4-PFDA	0.132	0.002	N/F	0.006	0.003	0.008	0.006
	H4-PFUnDA	0.067	N/F	N/F	N/F	N/F	N/F	N/F
	H4-PFDoDA	0.013	N/F	N/F	N/F	N/F	N/F	N/F
	H5-PFHpA	0.093	N/F	N/F	N/F	N/F	0.004	0.005
	H5-PFOA	0.016	N/F	N/F	N/F	0.002	N/F	N/F
	H5-PFNA	0.148	N/F	N/F	0.001	0.000	N/F	0.017

	H5-PFDA	0.008	N/F	N/F	N/F	N/F	N/F	N/F
	H5-PFUnDA	0.008	N/F	N/F	N/F	N/F	N/F	N/F
	H5-PFDoDA	0.003	N/F	N/F	N/F	N/F	N/F	N/F
	H5-PFTriDA	0.000	N/F	N/F	N/F	N/F	N/F	N/F
	H5-PFTeDA	0.000	N/F	N/F	N/F	N/F	N/F	N/F
	H5-PFPeDA	0.002	0.001	0.000	0.001	0.002	N/F	N/F
**Response factor**	Average IS response C_4-C_{12} PFCAs	***4.0E+07***	***6.5E+07***	***5.3E+07***	***5.1E+07***	***4.0E+07***	***3.5E+07***	***3.1E+07***

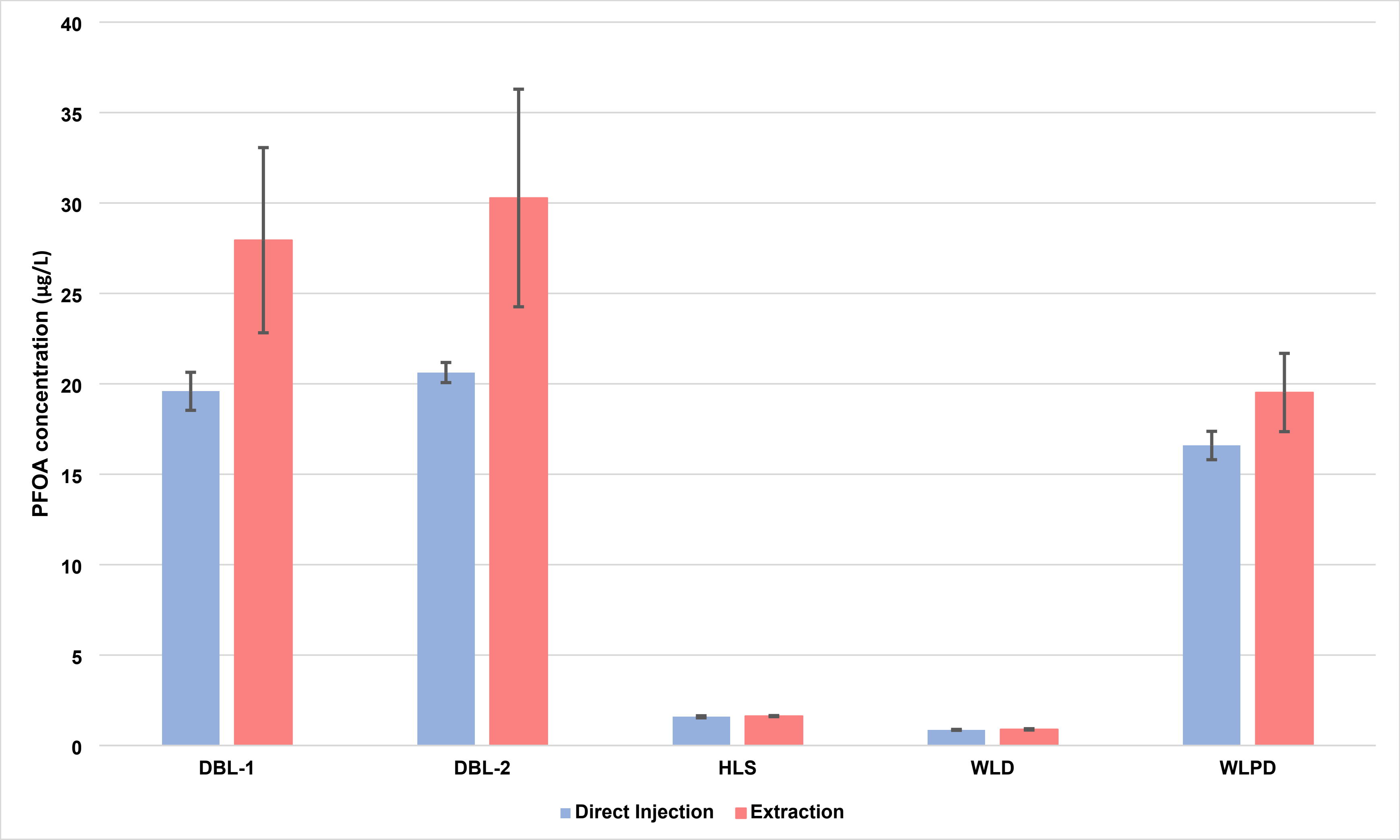

Figure S1. Comparison of PFOA concentrations determined by direct injection (blue) vs. SPE-extraction (red) in the samples with high PFOA responses; DBL = discharge by lagoon, HLS = Hillylaid Pool, WLD = Wyre land drain and WLPD = Wyre leaking pipe drain.

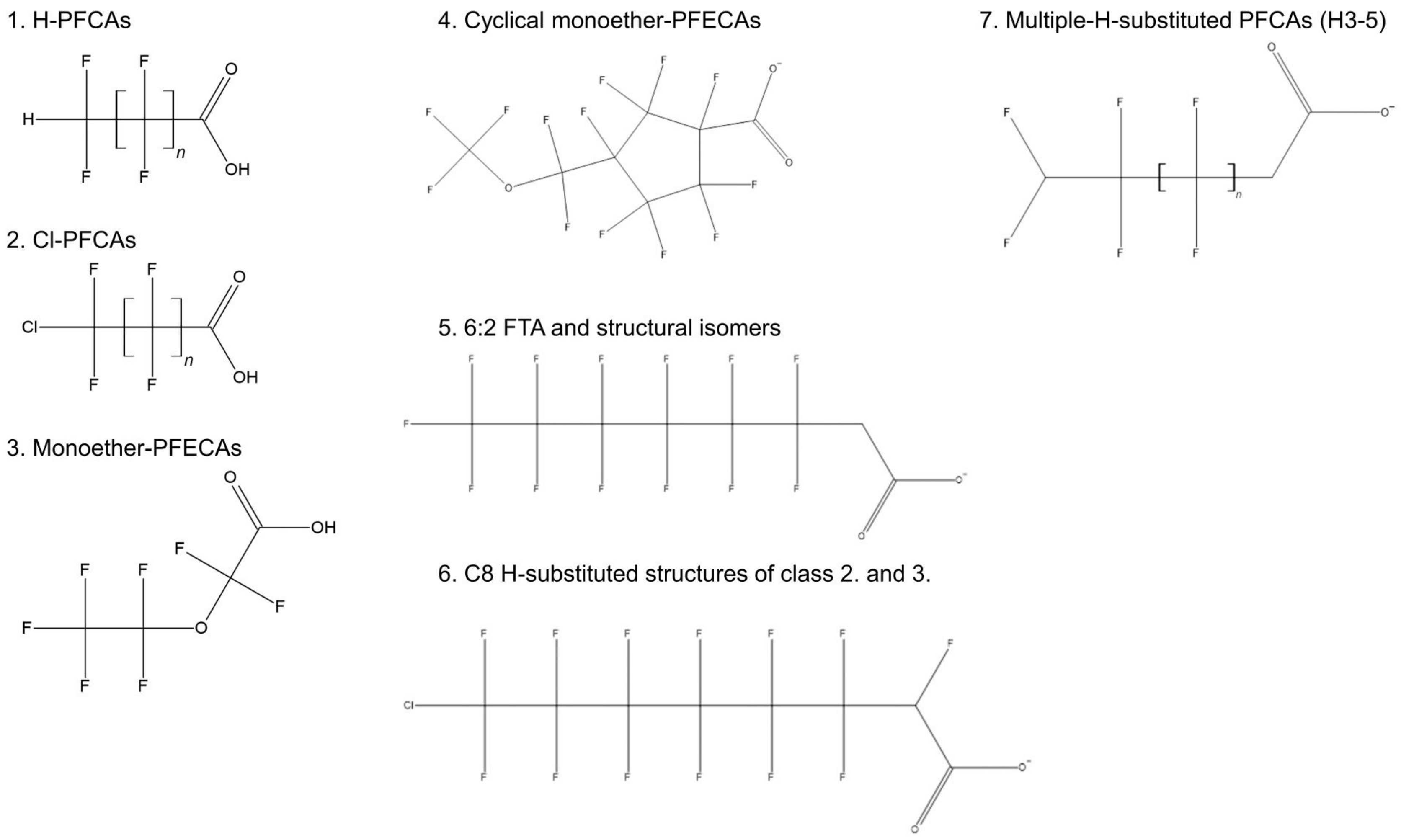

Figure S2. PFAS classes and homologs detected in the suspect screening. Note that the position of the H, Cl or ether-linkage for class 1., 2., 3., 5., 6. or 7. are tentative or could differ

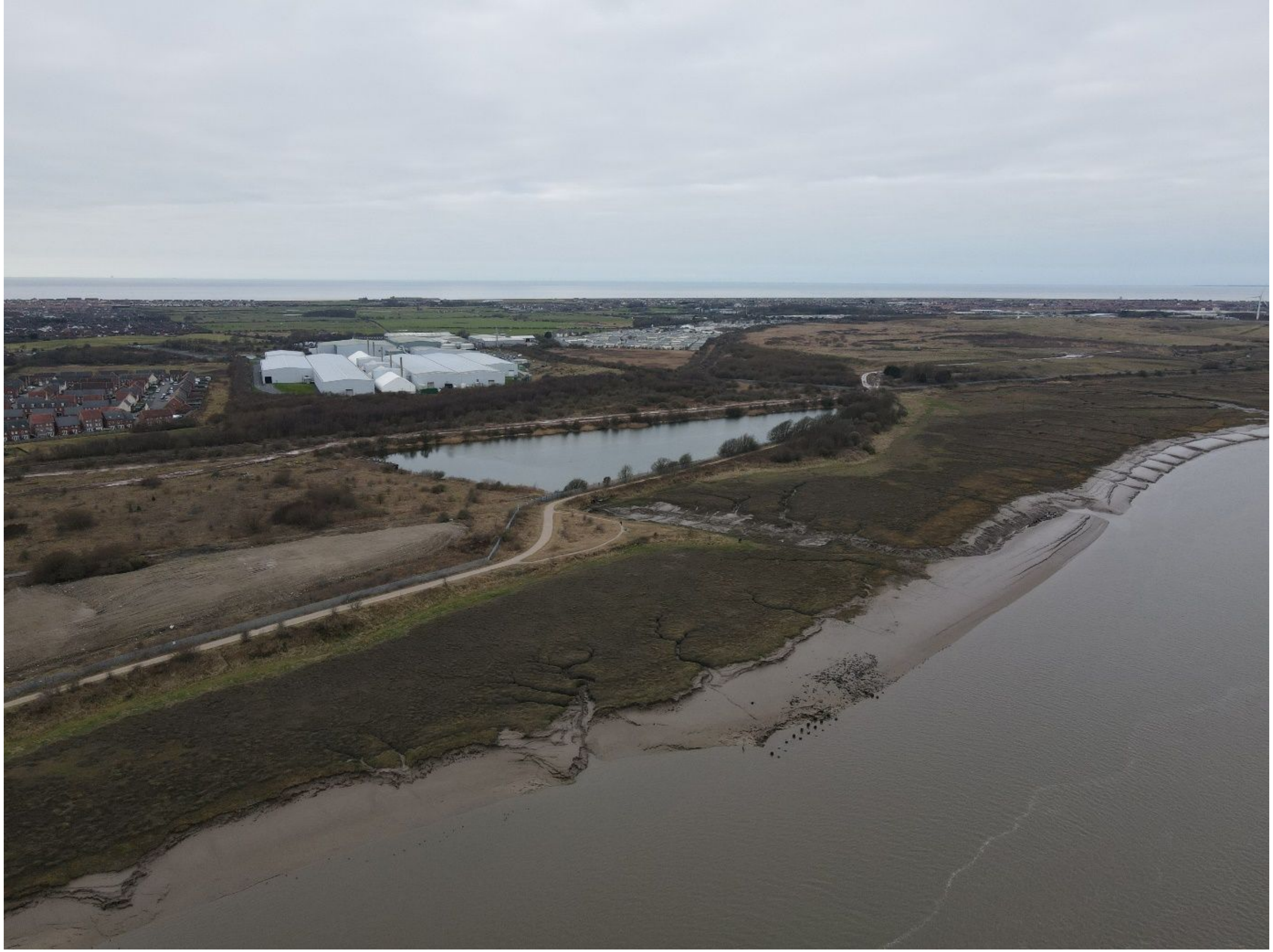

Figure S3. Photo of the lagoon and discharge to north of the plant (DBL). The landfill is on the background.

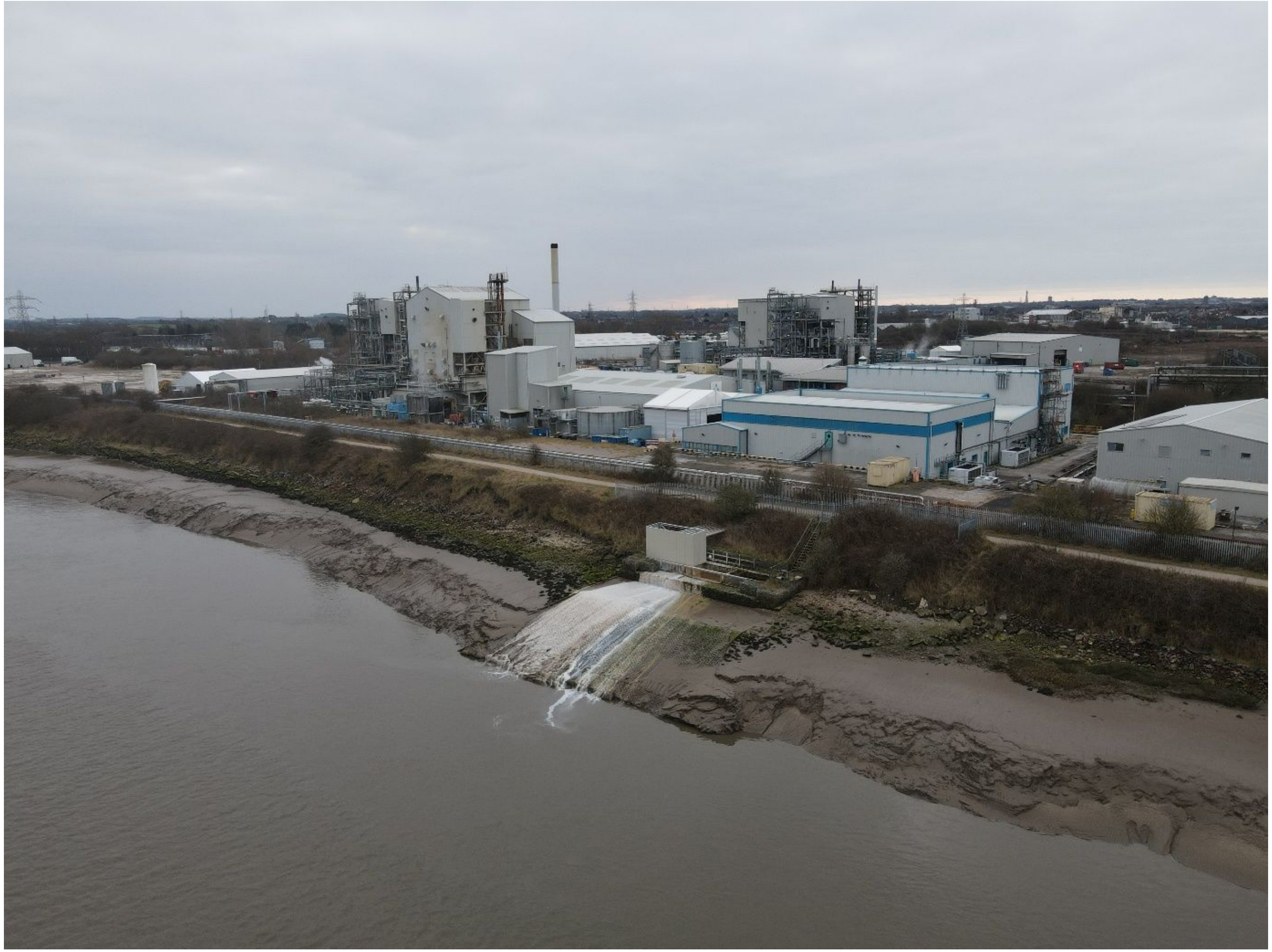

Figure S4. The main discharge at the River Wyre (WMD) with AGC Chemicals in the background. The plant in the foreground is the Victrex PEEK production plant

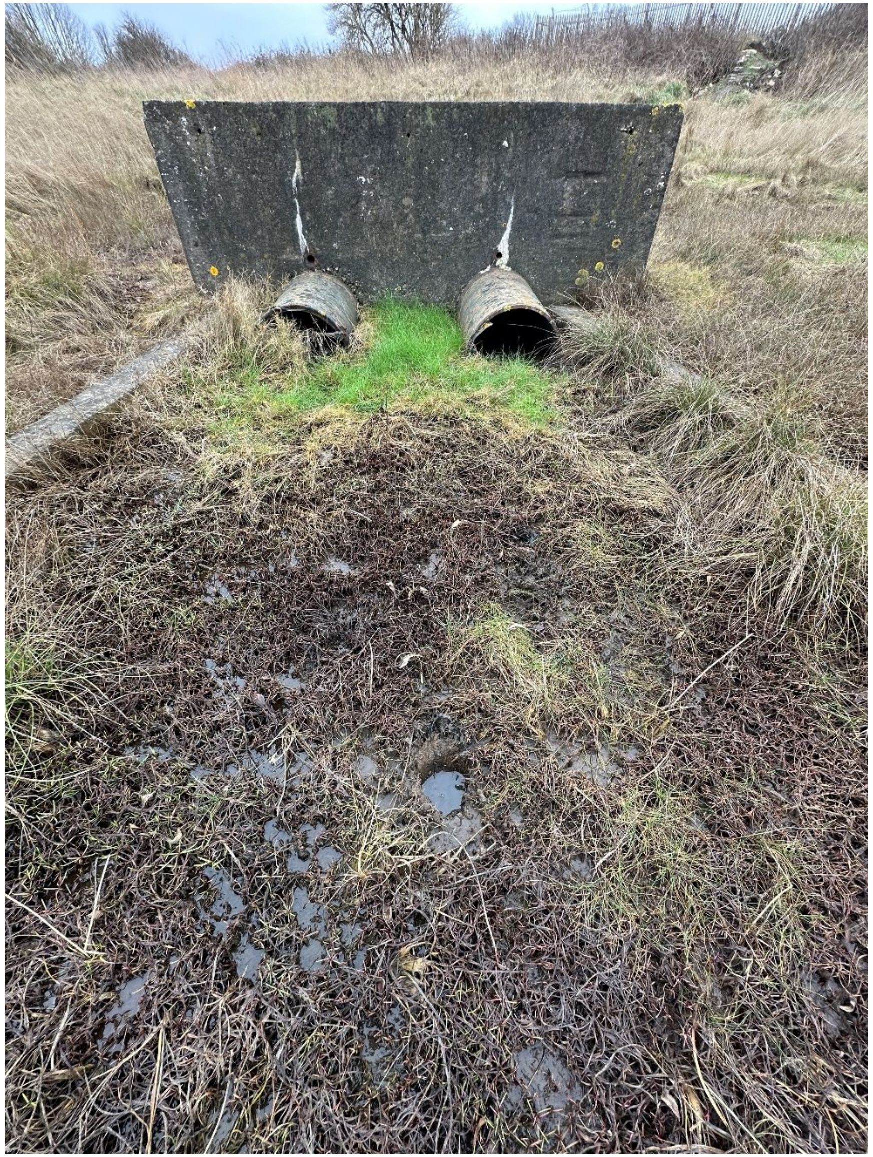

Figure S5. Land drain near the north of the lagoon (WLD)

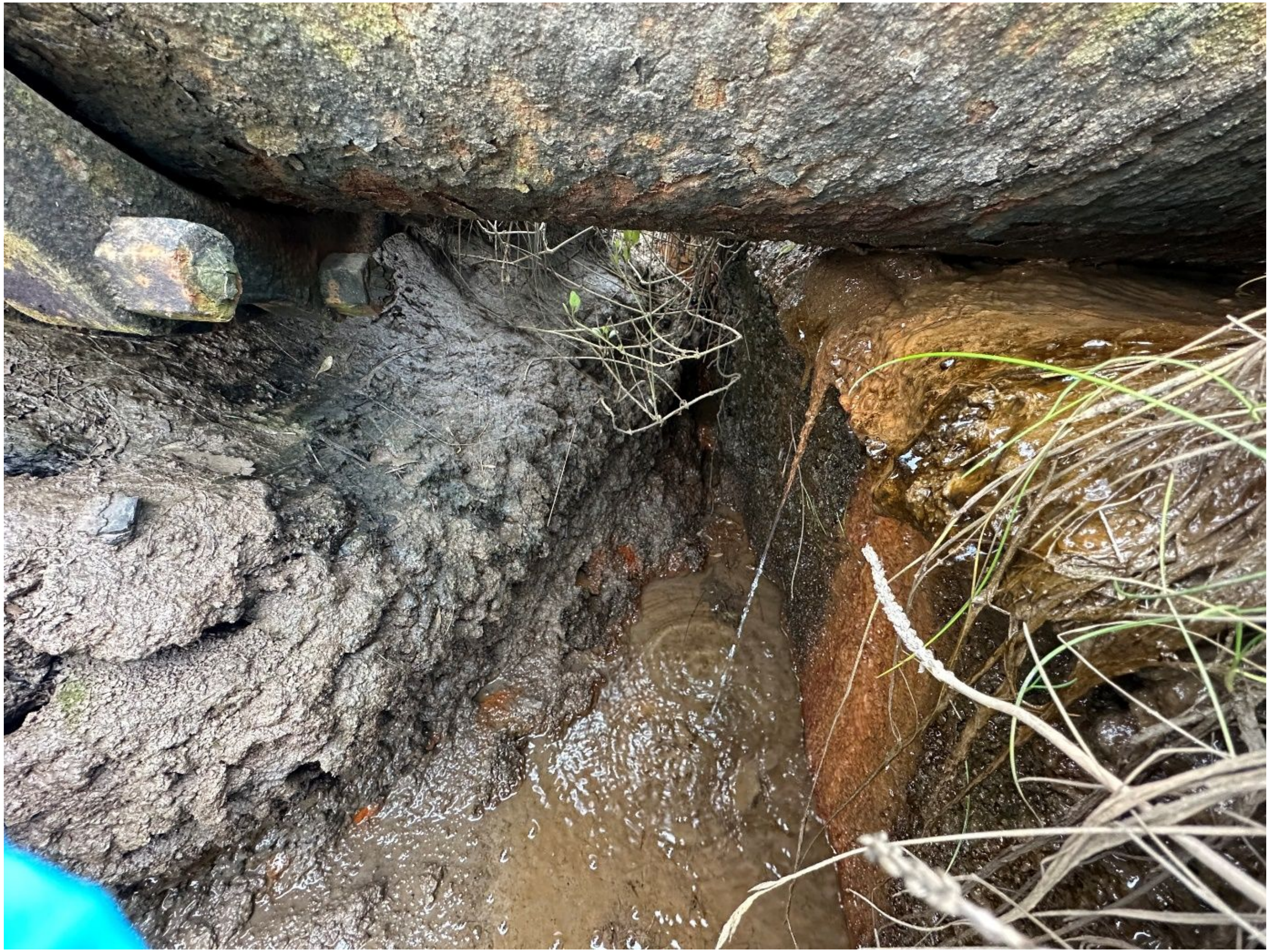

Figure S6. The leaking pipe to the north of the lagoon (WLPD)

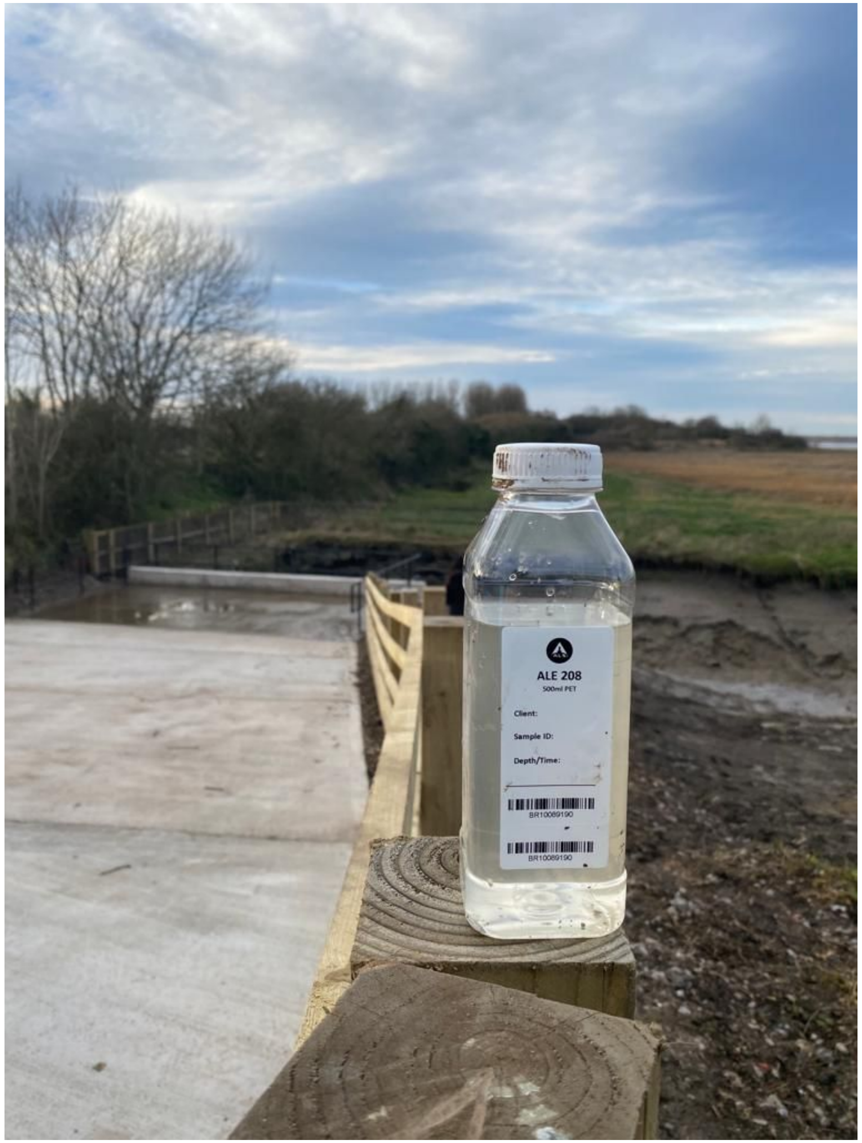

Figure S7. The Hillylaid Pool surface water discharge (HLP)

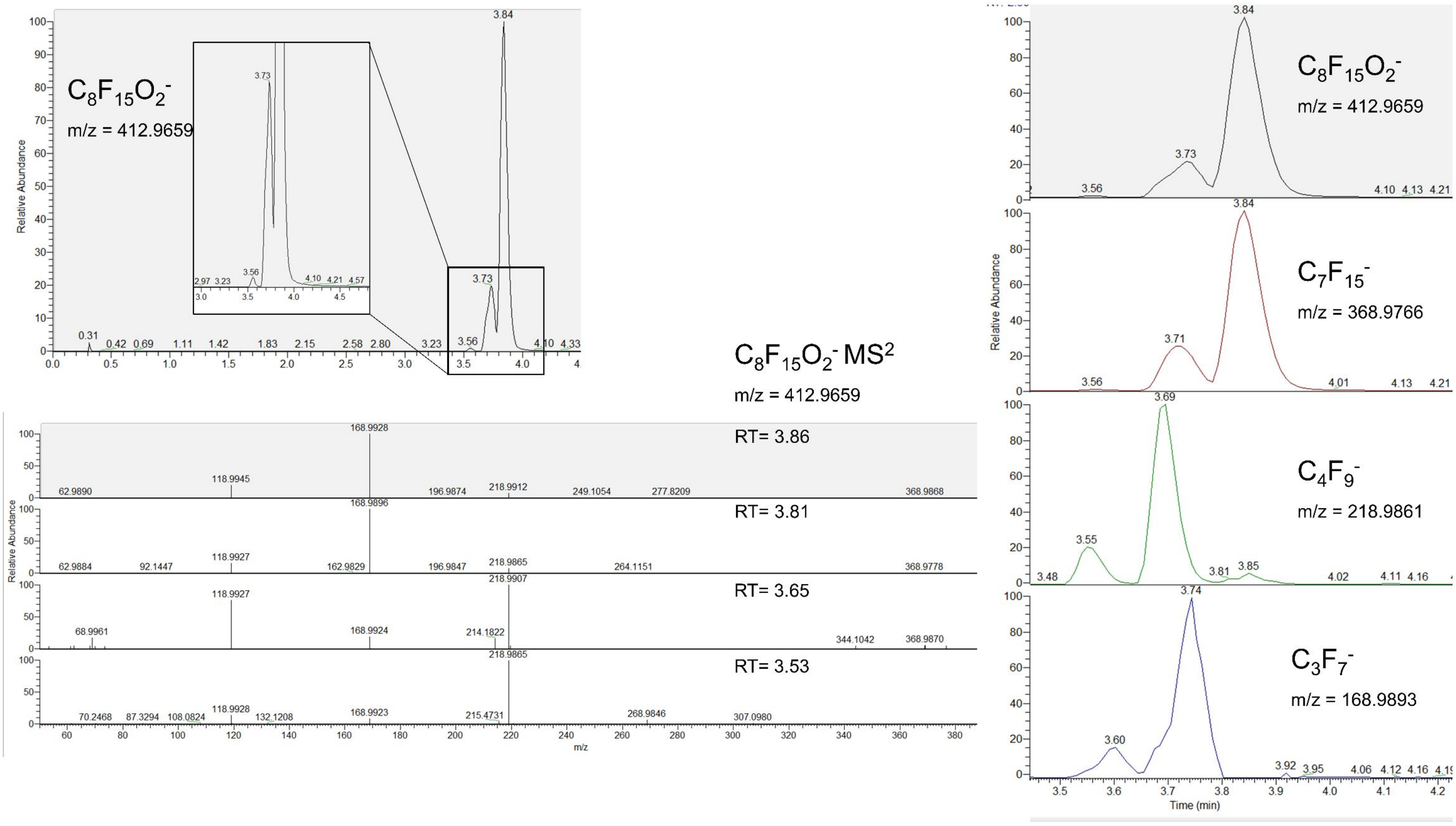

Figure S8. Identification of branched PFOA isomers

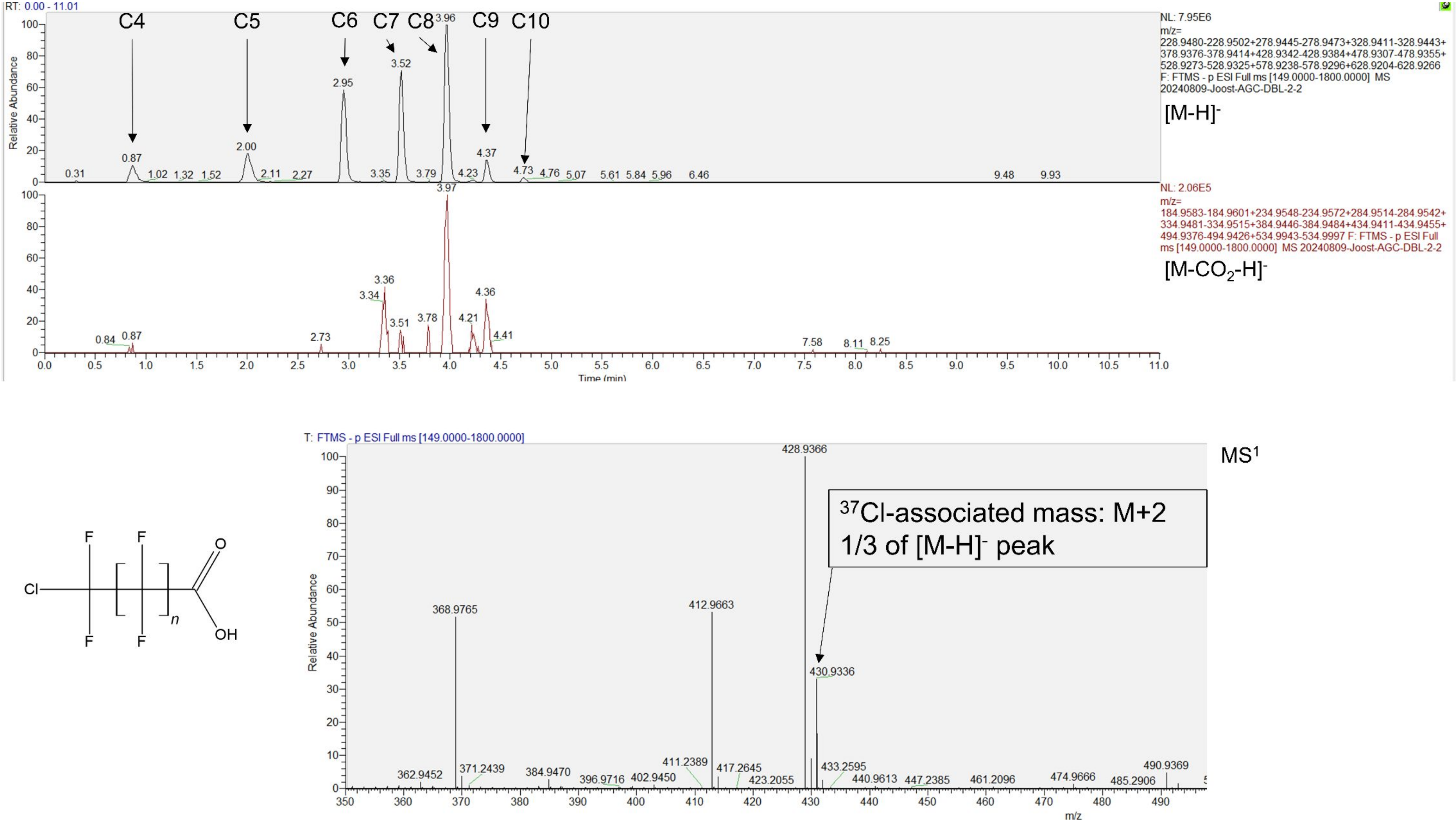

Figure S9. Identification of Cl-PFCA homolog series in the water samples

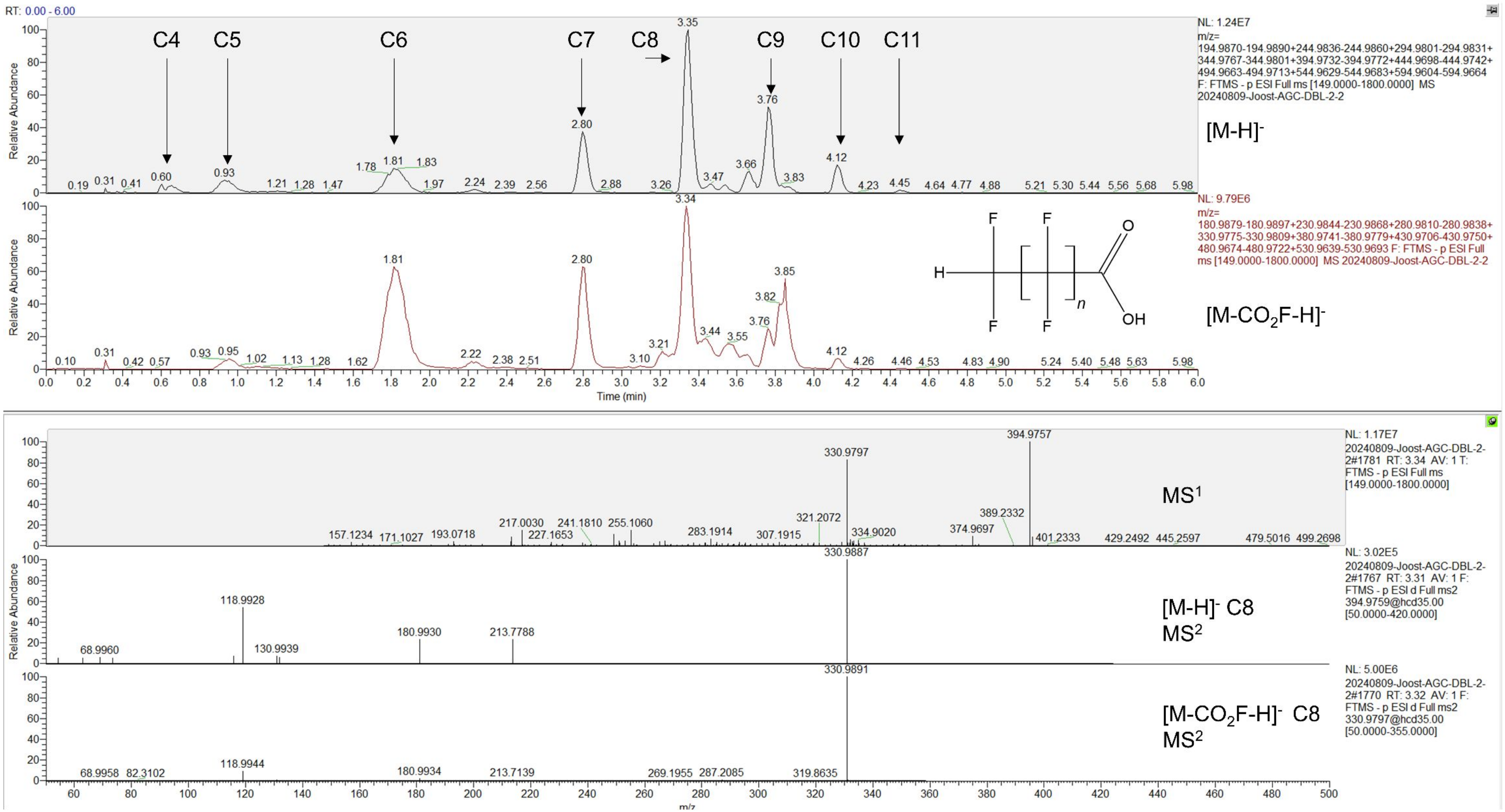

Figure S10. Identification of H-PFCA homolog series in water sample with example of spectra for the C8 homolog

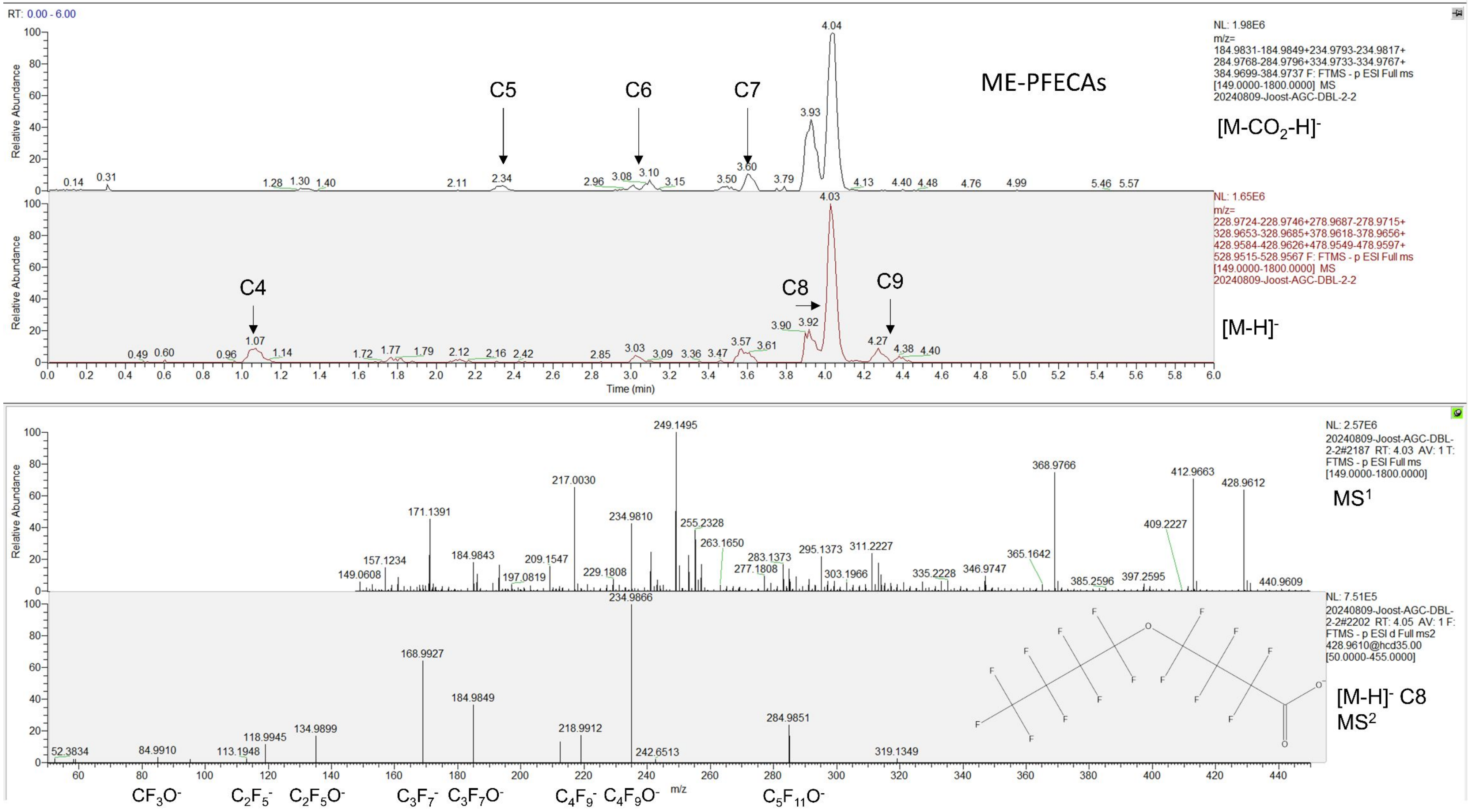

Figure S11. Identification of ME-PFECA homolog series in water sample with example of spectra for the C8 homolog

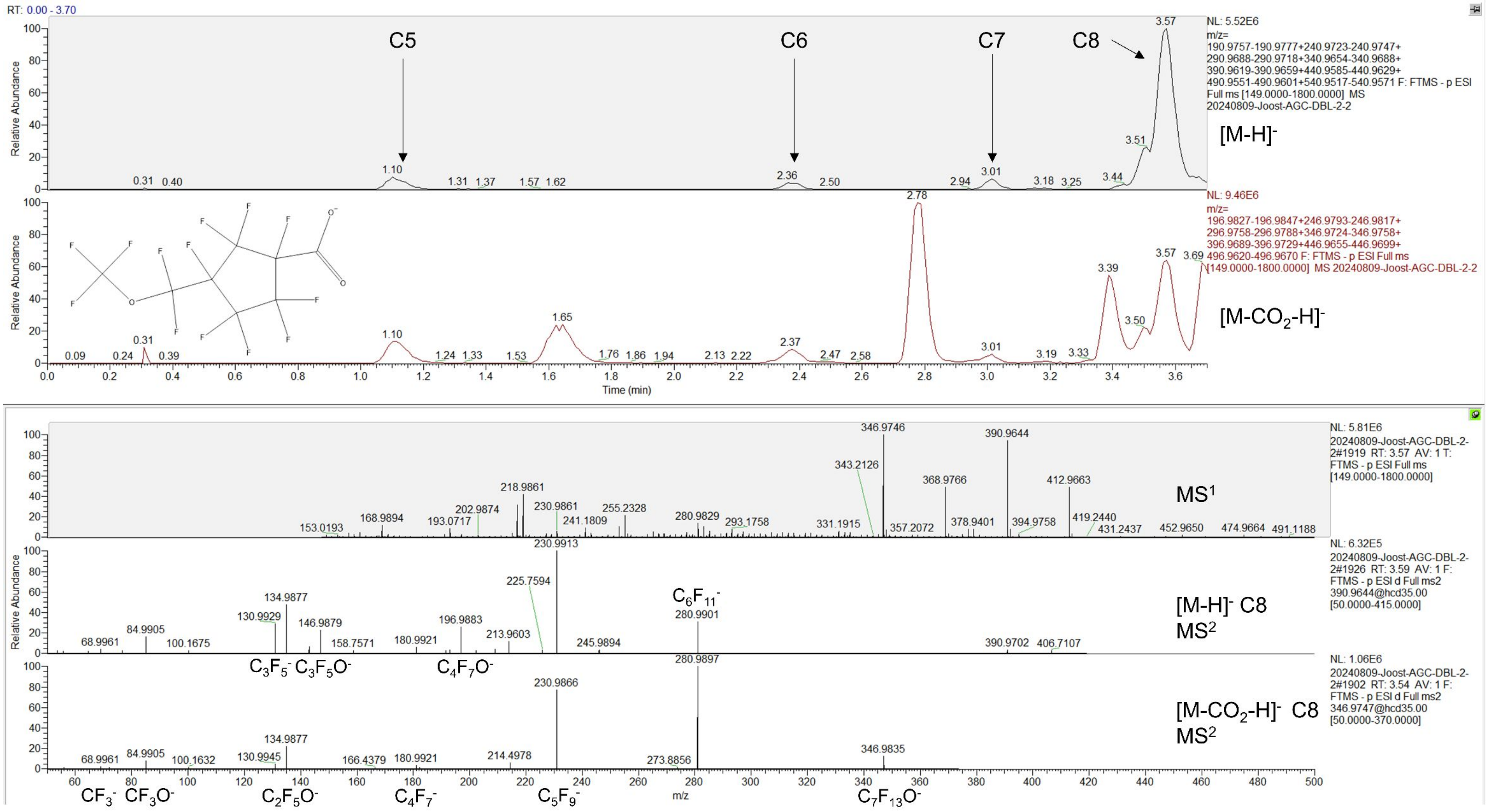

73

Figure S12. Identification of cyclical ether homolog series in water sample with example of spectra for the C8 homolog

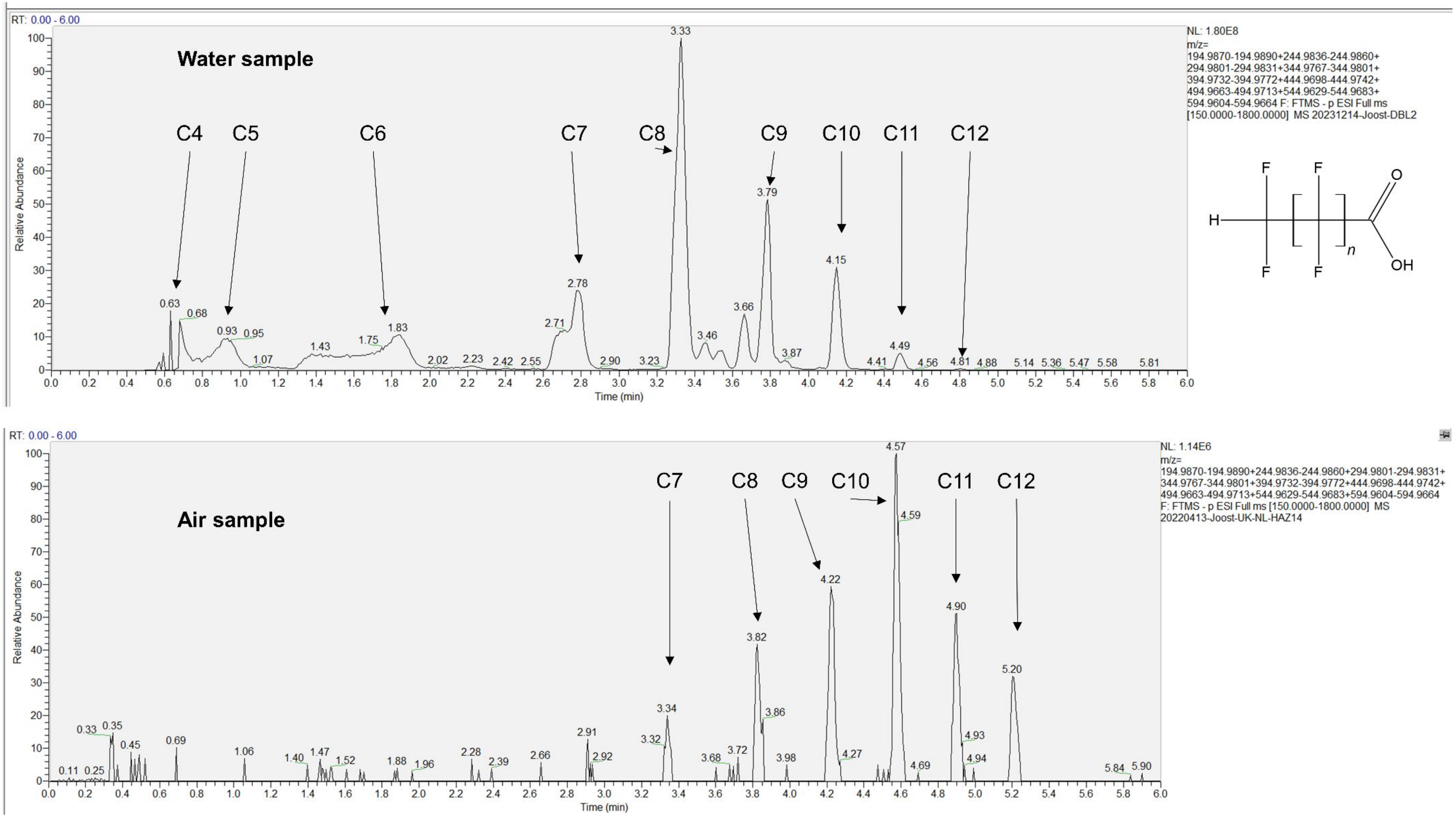

Figure S13. Larger relative area of longer-chained H-PFCA homologs in air sample compared to water sample

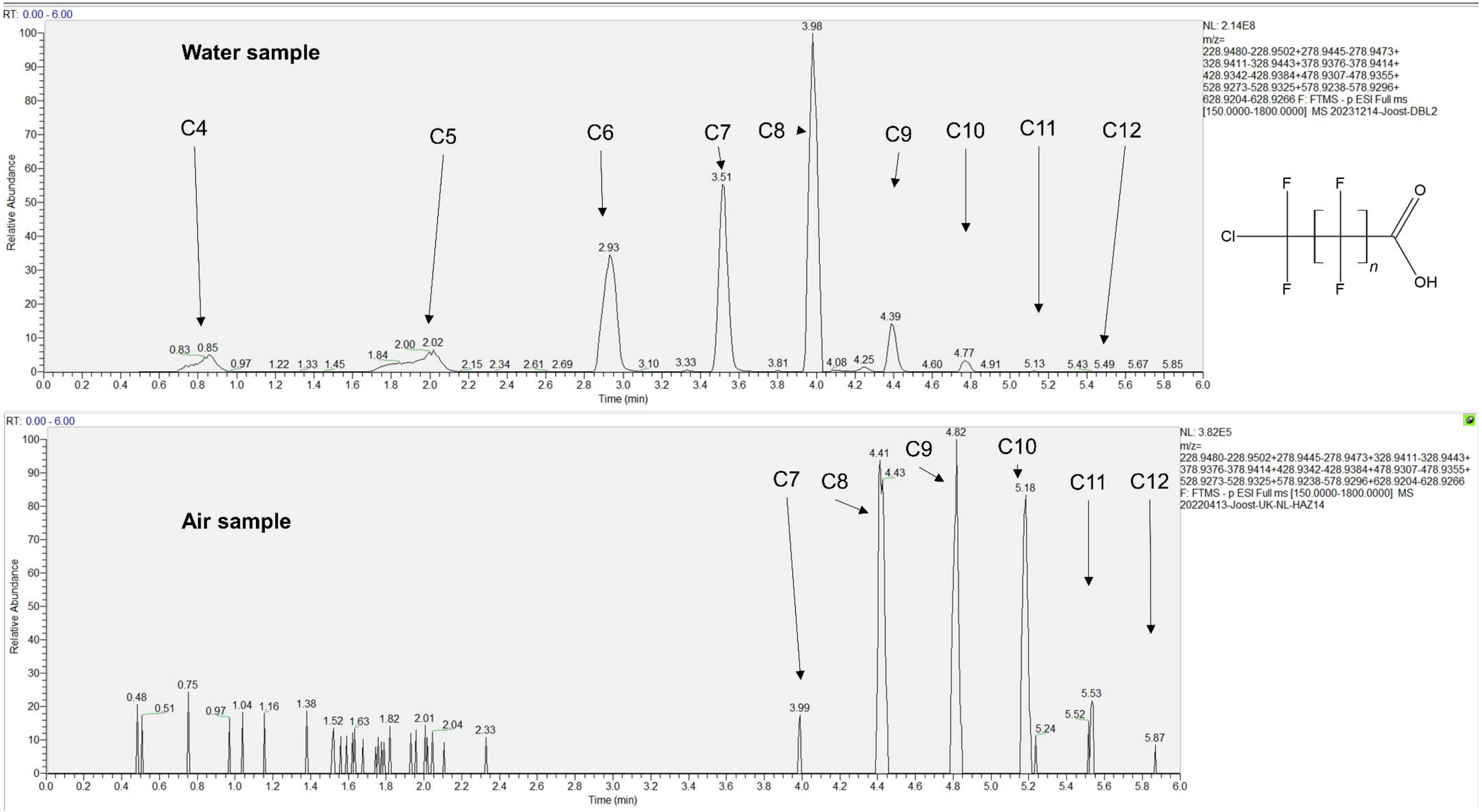

79

80 *Figure S14. Larger relative response of longer-chained Cl-PFCA homologs on the air sample compared to the water sample*

81

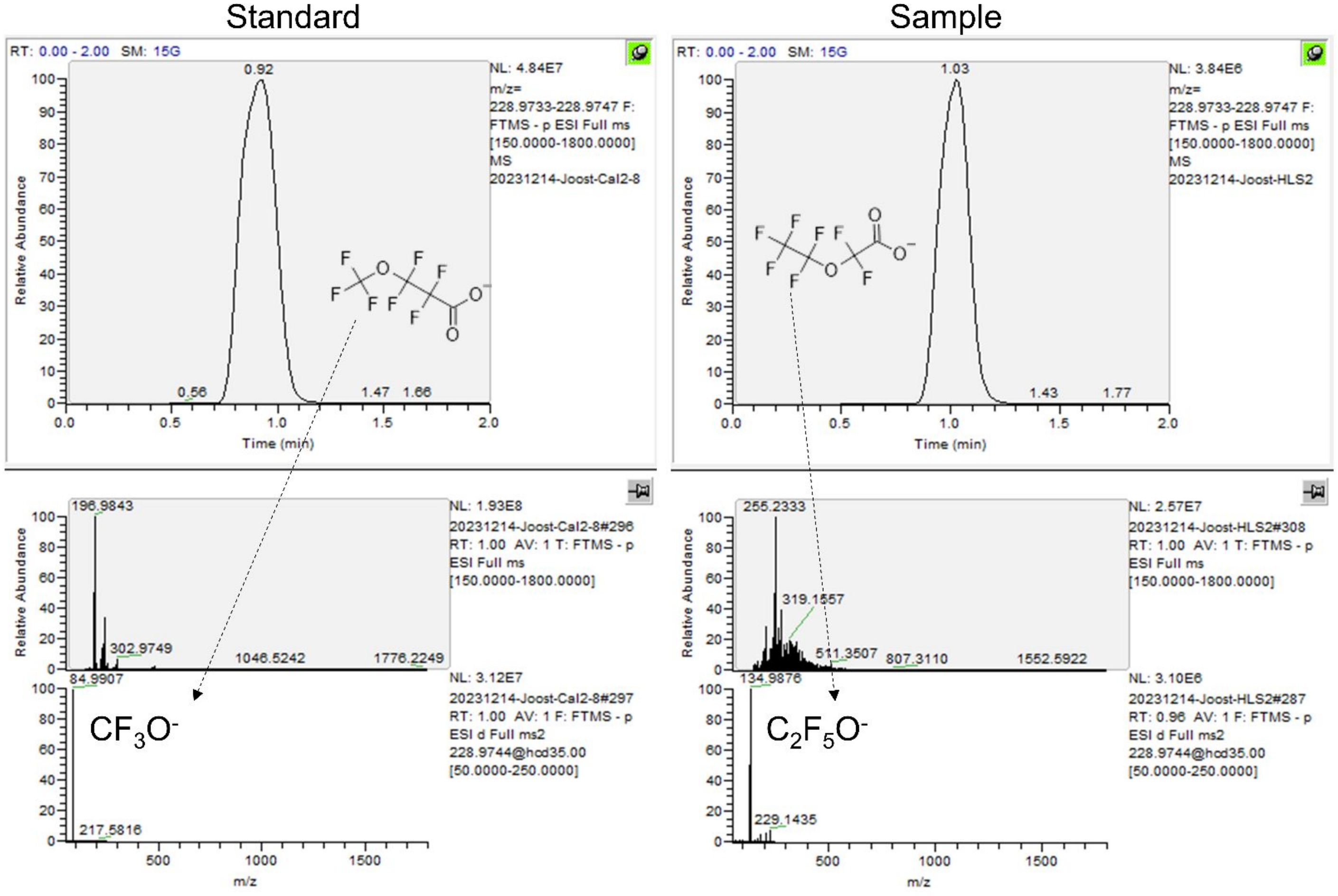

82

83 *Figure S15. Comparison between the ether PFBA measured in the standard and sample and isolated MS² fragments showing the substance*
84 *found in the sample is probably an isomer with the ether linkage at a different position.*

Wind direction analysis

Similar to previous work, [1] the following equations were used to calculate the average wind speed and direction during the sampling periods from hourly data:

1. $V_{East(i)} = mean(WS_{(i)} * sin(WD_{(i)} * \frac{\pi}{180}))$ (S1)

2. $V_{North(i)} = mean(WS_{(i)} * cos(WD_{(i)} * \frac{\pi}{180}))$ (S2)

3. $WD_{avg} = 360 + atan2(V_{East}, V_{North}) * \frac{180}{\pi}$ (°) (S3)
4. $WS_{avg} = \sqrt{(V_{East}{}^2 + V_{North}{}^2)}$ (ms^{-1}) (S4)

In which V is the vector of wind speed (WS, ms^{-1}), wind direction (WD, °) and atan2 is the 2-argument arctangent. Subsequently, the averaged wind speed and wind direction data, along with the PFAS air concentration data were used as input for the pollutionRose function of the openair R package [2] for plotting the pollution roses of the data.

Bin widths for the wind roses were determined by taking the square root of the number of samples > LOD and rounding up.

References

1. Dalmijn J, Shafer JJ, Benskin JP, Salter ME, Johansson JH, Cousins IT. HFPO-DA and Other PFAS in Air Downwind of a Fluoropolymer Production Plant in the Netherlands: Measurements and Modeling. *Environmental Science & Technology*. 2025 April.

2. Carslaw DC, Ropkins K. Openair—an R package for air quality data analysis. *Environmental Modelling & Software*. 2012;27: p. 52–61.
